# Predicting Tuberculosis Risk in Cattle, Buffaloes, Sheep, and Goats in China Based on Air Pollutants and Meteorological Factors

**DOI:** 10.3390/ani14243704

**Published:** 2024-12-22

**Authors:** Le Xu, Suya Li, Hong Li, Haoju Pan, Shiyuan Li, Yingxue Yang, Yuqing Jiao, Feng Lan, Si Chen, Qiaoling Chen, Li Du, Churiga Man, Fengyang Wang, Hongyan Gao

**Affiliations:** Hainan Key Laboratory of Tropical Animal Reproduction & Breeding and Epidemic Disease Research, School of Tropical Agriculture and Forestry, Hainan University, Haikou 570228, China; 22220952000023@hainanu.edu.cn (L.X.); 17638522327@163.com (S.L.); 20177304310011@hainanu.edu.cn (H.L.); haoju36999@163.com (H.P.); 22lishiyuan@hainanu.edu.cn (S.L.); 23220952000023@hainanu.edu.cn (Y.Y.); 23210905000003@hainanu.edu.cn (Y.J.); 23220952000014@hainanu.edu.cn (F.L.); chensi.ruth@hotmail.com (S.C.); chengiaoling1987@sina.cn (Q.C.); kych2008dl@163.com (L.D.); manchuriga@163.com (C.M.)

**Keywords:** bovine tuberculosis, sheep tuberculosis, goat tuberculosis, maximum entropy model, air pollutant, meteorological factor

## Abstract

Tuberculosis is a zoonotic chronic respiratory infectious disease caused by the *Mycobacterium tuberculosis* complex. The outbreak and epidemic of tuberculosis can seriously threaten human and veterinary health. To investigate the effects of environmental factors on tuberculosis in domestic ruminants, we collected data regarding the prevalence of tuberculosis in cattle, buffaloes, sheep, and goats in China (1956–2024) from publicly published literature and available databases. We identified the key risk factors among six major air pollutants and 19 bioclimatic variables; simulated the risk distribution of tuberculosis in cattle, buffaloes, sheep, and goats in China using the maximum entropy ecological niche model; and evaluated the effects of environmental factors. The area under the curve of the model was 0.873 (95% confidence interval, 0.851–0.895). The risk factors that most significantly influenced the prevalence of tuberculosis were the nitrogen dioxide (NO_2_) level, mean temperature of the coldest quarter, cattle distribution density, sheep distribution density, ozone (O_3_) level, and precipitation of the driest month. The predicted map of tuberculosis risk in cattle, buffaloes, sheep, and goats indicated that the high-risk regions were mainly distributed in South, North, East, and Northwest China. Improved surveillance is needed in these high-risk areas, and early preventive measures must be implemented based on the risk factors identified to reduce the future prevalence of tuberculosis in cattle, buffaloes, sheep, and goats.

## Simple Summary

To investigate the effects of air pollutants and bioclimatic variables on tuberculosis in domestic ruminants, we simulated the risk distribution of tuberculosis in cattle, buffaloes, sheep, and goats in China using the maximum entropy ecological niche model, and evaluated the effects of environmental factors. The risk factors that most significantly influenced the prevalence of tuberculosis were the nitrogen dioxide (NO_2_) level, mean temperature of the coldest quarter, cattle distribution density, sheep distribution density, ozone (O_3_) level, and precipitation of the driest month. The predicted map of tuberculosis risk in cattle, buffaloes, sheep, and goats indicated that the high-risk regions were mainly distributed in South, North, East, and Northwest China. The findings of this study provide a valuable reference for the prevention and control of tuberculosis in cattle, buffaloes, sheep, and goats in China.

## 1. Introduction

The advancement of global industrialization and urbanization has led to the exacerbation of air pollution caused by emissions from urban transportation and various industrial activities. The levels of air pollutants, such as ammonia, methane, hydrogen sulfide, sulfur dioxide (SO_2_), carbon dioxide, carbon monoxide (CO), and particulate matter (including PM_2.5_ and PM_10_), are increasing annually [1,2,3]. Studies have shown that air pollutants can reduce the immune resistance in humans and animals, thereby inducing a range of inflammatory conditions or complications [4,5]. In addition, air pollutants and meteorological factors substantially contribute to the exacerbation and pathogenesis of chronic respiratory diseases in humans [6,7].

Tuberculosis is a zoonotic chronic respiratory disease caused by the *Mycobacterium tuberculosis* complex (MTBC), which primarily consists of *M. tuberculosis*, *M. bovis*, *M. africanum*, and *M. caprae* [8]. Of these, *M. tuberculosis* and *M. bovis* have the widest host range and can infect humans and other animals [9,10,11]. Tuberculosis can spread to various animals, including domestic ruminants, poultry, and humans, posing a major threat to their health. According to the 2022 Global Tuberculosis Report (https://www.who.int/, accessed on 11 January 2024), approximately 10.6 million people suffer from tuberculosis globally, resulting in approximately 1.3 million deaths. Furthermore, bovine tuberculosis is a notifiable animal disease as per the Office International des Épizooties.

Domestic ruminants infected with tuberculosis serve as potential sources of tuberculosis and act as transmission vectors between animals and humans [12]. China boasts a large breeding scale of ruminants. According to the National Bureau of Statistics (https://www.stats.gov.cn, accessed on 20 August 2024), the number of cattle in China reached 105.085 million heads in 2023, whereas that of sheep and goats reached 322.326 million heads, indicating the largest population of these ruminants worldwide. The presence of multiple hosts and the possibility of interspecies transmission make the prevention and control of tuberculosis extremely challenging. However, no effective vaccine or treatment has been developed for tuberculosis in cattle, buffaloes, sheep, or goats [13]. Once a disease appears, it is usually controlled through culling. Tuberculosis directly impacts the development of livestock production and economic growth as well as veterinary public health safety.

Researchers have conducted several investigations into the risk factors of tuberculosis to further understand the mechanisms underlying the transmission and occurrence of tuberculosis. Several studies have shown that air pollution can increase the prevalence of tuberculosis [14,15]. Furthermore, meteorological factors may affect the level and composition of air pollutants [16,17,18]. Thus, the effects of environmental factors, such as temperature and precipitation, should be considered when studying the risk factors for tuberculosis. The factors contributing to tuberculosis are complex and varied, and the effect of environmental factors on the prevalence of animal tuberculosis remains unclear. This study is the first to investigate the effects of air pollution and meteorological conditions on tuberculosis in domestic ruminants. Furthermore, most existing studies have used statistical analysis methods such as time series analysis, generalized additive models, Spearman correlation analysis, and penalized distributed lag nonlinear models [19,20,21,22], which cannot predict the risk distribution of tuberculosis or provide recommendations on regional prevention and control. This study used the maximum entropy (MaxEnt, version 3.4.1) algorithm to construct an ecological niche model that can intuitively display the probability of disease occurrence. MaxEnt is a robust machine learning algorithm that can be used to predict potential spatial distributions based on the presence of species loci and environmental variables. Owing to its excellent predictive performance and early warning features, the model has been widely used to predict high-prevalence areas of animal diseases [23,24].

This study explored the risk distribution of tuberculosis in cattle, buffaloes, sheep, and goats in China and investigated the potential effects of various air pollutants and meteorological factors on the tuberculosis risk, with a view to more effectively guiding the implementation of tuberculosis prevention and control measures.

## 2. Materials and Methods

### 2.1. Occurrence Data

This study identified 334 tuberculosis occurrence sites from 1956 to 2024, of which 324 sites were from cattle, 7 sites were from buffalo, 1 site was from goats, and 2 sites were from sheep (Table S1). The data were obtained from the China National Knowledge Infrastructure, VIP Database for Chinese Technical Periodicals, Wanfang Database, PubMed, the Web of Science, and other databases. For records that contained specific geographic details but no coordinates, we used Baidu Maps (https://api.map.baidu.com/, accessed on 3 January 2024) to obtain the coordinates.

To reduce the spatial autocorrelation and eliminate duplicates, data within a 1 km range were considered as only one instance. In total, 264 records of tuberculosis in cattle, buffaloes, sheep, and goats were obtained (Table S2).

### 2.2. Environmental Variables

To establish a MaxEnt model of tuberculosis in cattle, buffaloes, sheep, and goats, a total of 29 environmental variables were considered (Table 1).

(1) Nineteen bioclimatic variables (Bio 1–Bio 19) representing the global climate conditions (temperature and precipitation) were downloaded from WorldClim (https://worldclim.org/data/worldclim21.html#, accessed on 21 June 2024).
(2) Six common air pollutants, namely PM_2.5_, PM_10_, CO, NO_2_, SO_2_, and O_3_, were downloaded from Zenodo (https://zenodo.org/communities/chap, accessed on 20 May 2024) to obtain raster maps. The Raster Calculator in ArcToolbox (version 2.5) was used to calculate their average raster values.
(3) Distribution density maps of four livestock species (cattle, buffaloes, sheep, and goats) were downloaded from the Food and Agriculture Organization of the United Nations (http://www.fao.org, accessed on 25 June 2024).

As multicollinearity between the bioclimatic variables can cause overfitting of the model predictions [25], the multicollinearity between the 19 bioclimatic variables and six air pollutants was excluded before modeling. Since there was no linear relationship between the variables, a correlation analysis was conducted between the two types of variables using the Spearman correlation coefficient (r). A strong correlation was indicated when |r| was ≥0.7 [26]. Then, we established a pre-model and removed the variables with smaller contribution rates from the two strong correlation variables. Finally, we standardized the variables included in the model to a consistent spatial range and converted them to ASCII format via ArcGIS 10.8. These variables were then used for modeling.

### 2.3. Establishment of the MaxEnt Ecological Niche Model

The working principle of the MaxEnt model is to maximize the entropy (uncertainty) and derive a probability function corresponding to the distribution of the known presence points to ensure uniform probability distribution under certain conditions. The mathematical formula of the MaxEnt model is as follows [27,28]:

(1) $$P_{\omega }\left(y|x\right)=\frac{1}{Z_{\omega }\left(x\right)}exp\left(\sum_{i=1}^{n}\omega ifi\left(x|y\right)\right)$$

(2) $$Z_{\omega }\left(x\right)=\sum_{y}exp\left(\sum_{i=1}^{n}\omega ifi\left(x|y\right)\right)$$

where x represents the environmental variable input into the model; y represents the predicted geographical area; $fi\left(x|y\right)$ represents the feature functions; ωi represents the weights associated with $fi\left(x|y\right)$; and $Z_{\omega }\left(x\right)$ indicates the normalization constant.

Thus, the MaxEnt model provides a good assessment of the temporal and spatial patterns of disease burden. We used MaxEnt software (version 3.4.1) to construct the tuberculosis risk model. To evaluate the predictive accuracy of the model, 25% of the tuberculosis data were randomly selected by the program as test samples. The remaining 75% of the tuberculosis data were used as training samples for the model construction. To minimize the sampling bias, we selected 10,000 background points as the “pseudo-absent” data. The model was run 10 times, and the average value of the 10 runs was used as the final result.

We evaluated the performance of the model by constructing a receiver operating characteristic curve and evaluating the area under the curve (AUC) [29]. The AUC values ranged from 0 to 1. The closer the AUC value is to 1, the better the model’s ability to classify, while an AUC value close to 0.5 indicates that the model’s performance is comparable to random speculation.

ArcGIS v10.8 was used to visualize the potential risk map of tuberculosis.

## 3. Results

### 3.1. Variables Included in the Tuberculosis Model

**Figure 1:**
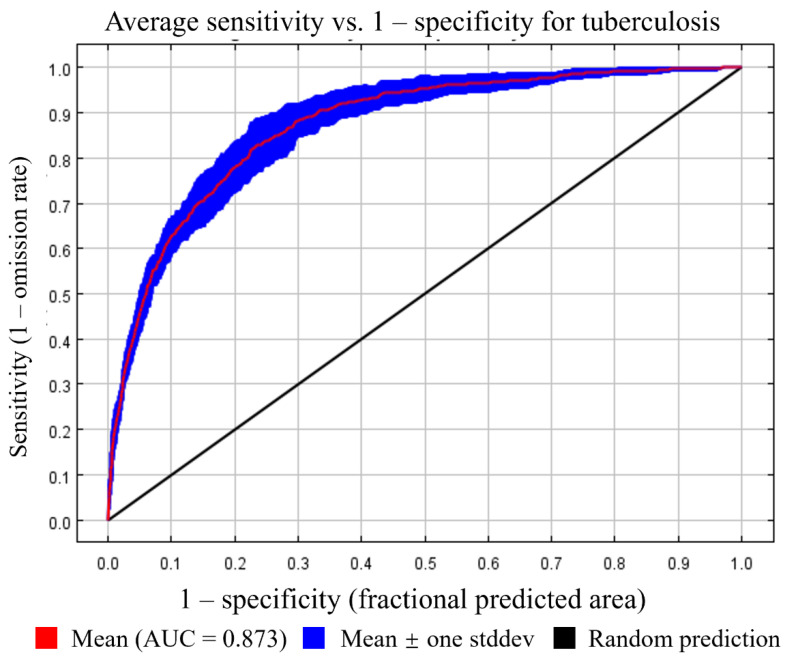
Receiver operating characteristic curves for a model of tuberculosis in cattle, buffaloes, sheep, and goats.

Spearman correlation analysis revealed a strong correlation between PM_2.5_ and PM_10_. A previous study showed that PM_10_ is more representative [30]; hence, the variable PM_2.5_ was removed from the model. The final tuberculosis model included five bioclimatic variables, five air pollutant variables, and four host distribution density variables (Table 1). Ultimately, the tuberculosis model demonstrated strong performance (Figure 1), with an average AUC value of 0.873 and a standard deviation of 0.022.

Table 2 presents the percentage contribution of each variable. The important variables that influenced the prevalence of tuberculosis were NO_2_ level, mean temperature of the coldest quarter, cattle distribution density, sheep distribution density, O_3_ level, and precipitation of the driest month, showing a cumulative contribution rate of 76.9%.

**Figure 2:**
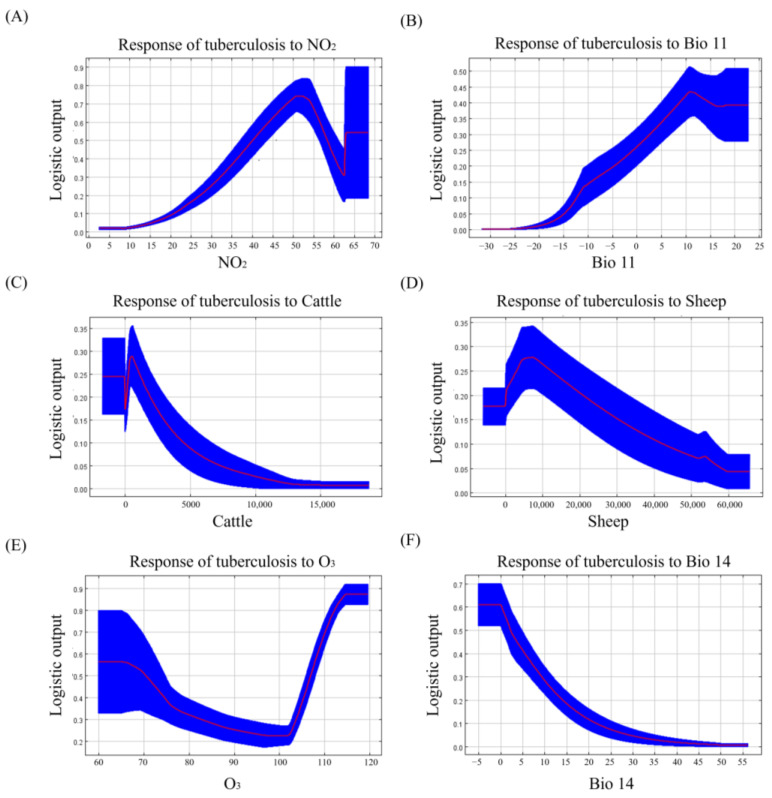
Response curves for important variables in a model of tuberculosis in cattle, buffaloes, sheep, and goats. Response curves for (**A**) nitrogen dioxide (NO_2_) level; (**B**) mean temperature of the coldest quarter (Bio 11); (**C**) cattle distribution density (Cattle); (**D**) sheep distribution density (Sheep); (**E**) O_3_ Level; and (**F**) precipitation of the driest month (Bio 14). Red is the response curve, blue is the standard error.

Figure 2 presents the response curves for the important variables in the tuberculosis model. The results showed that when the NO_2_ level ranged from 2.5 to 50.8 μg/m^3^, it was positively correlated with the prevalence of tuberculosis (Figure 2A). Similarly, the mean temperature of the coldest quarter was overall positively correlated with the prevalence of tuberculosis (Figure 2B). The response curves for the cattle and sheep distribution densities showed that within a specific area, the prevalence of tuberculosis increased with an increase in animal density. However, when the number of animals exceeded the carrying capacity of the area, the prevalence of tuberculosis showed a downward trend (Figure 2C,D). When the O_3_ ranged from 64.8 to 101 μg/m^3^, the prevalence of tuberculosis gradually decreased with increasing O_3_ level; however, when the O_3_ level ranged from 101 to 115 μg/m^3^, the prevalence of tuberculosis gradually increased with increasing O_3_ level (Figure 2E). Furthermore, the response curve for the precipitation of the driest month indicated that the prevalence of tuberculosis gradually decreased with increasing precipitation (Figure 2F).

### 3.2. Potential Risk Areas of Tuberculosis in Domestic Ruminants

**Figure 3:**
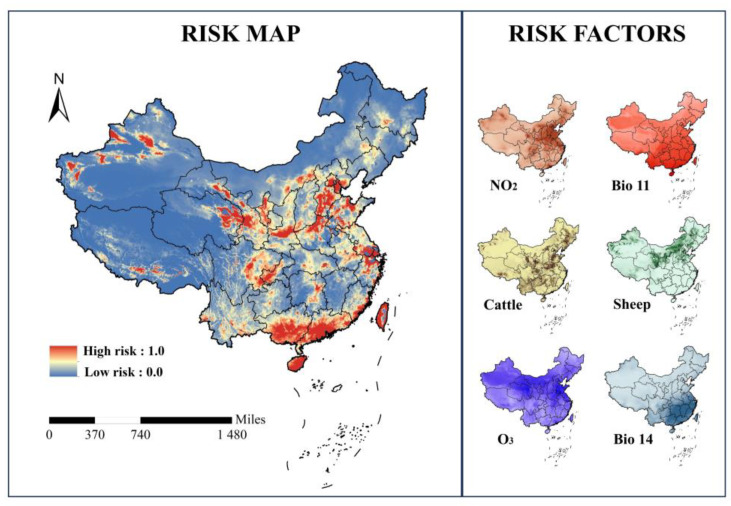
Potential risk map (**left**) and risk factors (**right**) of tuberculosis in cattle, buffaloes, sheep, and goats in China. This standard map of China can be downloaded from the standard map service system provided by the Ministry of Natural Resources.

Figure 3 shows the predicted risk areas of tuberculosis among domestic ruminants in China. The high-risk areas were primarily located in the northern Taklimakan Desert in Xinjiang; Guangxi Province; Guangdong Province; Hainan Province; Eastern Qinghai Province; parts of Shandong Province; Gansu Province; Eastern Sichuan Province; Central Shaanxi Province; Central Inner Mongolia; Beijing–Tianjin–Hebei region; Ningxia Hui Autonomous Region; Northern Henan Province; the border area between Jiangsu, Zhejiang, and Shanghai; and Taiwan Island.

## 4. Discussion

### 4.1. Analysis of the Important Risk Factors

A MaxEnt model was established based on air pollutant variables, bioclimatic variables, and livestock distribution density variables to predict the risk areas of tuberculosis among cattle, buffaloes, sheep, and goats in China. The model showed good performance. Previous studies have established an association between the prevalence of tuberculosis and level of air pollutants, especially NO_2_, PM_10_, and O_3_ [15,31]. The current study revealed that the NO_2_ level was the most important risk factor, and a NO_2_ level in the range of 2.5–50.8 μg/m^3^ was significantly positively correlated with the prevalence of tuberculosis. Several studies in China have shown that the prevalence of tuberculosis increases with rising NO_2_ levels [32,33]. Related studies in the United States and the European Union also found that NO_2_ exposure was positively correlated with the prevalence of tuberculosis [34,35]. An investigation of the pathogenic mechanism of NO_2_ revealed that NO_2_ is highly soluble in water, and when inhaled, it can induce inflammatory responses that further damage the mucous membranes, facilitating the invasion of *M. tuberculosis* into the lungs [36]. Furthermore, NO_2_ can weaken the immune system by inducing the production of tumor necrosis factor alpha (TNF-α) and interferon gamma (IFN-γ), thereby increasing the susceptibility to tuberculosis [37]. With the progressive implementation of air pollution control measures, the NO_2_ levels have gradually decreased over the years, which may mitigate the spread of tuberculosis in China.

O_3_ is another significant air pollutant that impacts the prevalence of tuberculosis. The response curve for the O_3_ level showed that the prevalence of tuberculosis increased initially and then decreased with increasing O_3_ levels. Some studies have shown that the O_3_ level is negatively correlated with the prevalence of tuberculosis [38,39]. However, some studies have indicated that elevated O_3_ levels increase the prevalence of tuberculosis [34]. whereas other studies have shown that the O_3_ level does not significantly affect the prevalence of tuberculosis [40,41]. These inconsistent results may be attributed to the uncertainty in the measurement of individual O_3_ exposure levels across various epidemiological studies. In terms of the potential physiological mechanisms, O_3_, a strong oxide, can rapidly and completely react with the surface of the respiratory tract after its entry into the lungs. Furthermore, the proinflammatory factors and oxidation products produced in the lungs can enter the blood circulation to induce systemic inflammatory and oxidative stress [42].

Among the meteorological factors, the response curve for the mean temperature of the coldest quarter indicated a positive correlation between temperature and the prevalence of tuberculosis. Research has shown that elevated temperatures boost pathogen replication and enhance their viability [43]; thus, high temperatures increase the prevalence of tuberculosis [44,45,46]. High temperatures also accelerate air circulation, which is conducive to the spread of pathogens, thereby increasing the risk of tuberculosis exposure [47,48]. The response curve for the precipitation of the driest month indicated that the prevalence of tuberculosis in cattle, buffaloes, sheep, and goats was negatively correlated with precipitation, as confirmed by some studies [49,50]. This finding may be attributed to increased rainfall, which reduces the concentration of dust or PM in the air, thereby reducing the probability of its attachment to pathogens [51]. Furthermore, the temperature and relative humidity significantly influence the determination of the droplet diameter [52], and the spread of *M. tuberculosis* is closely related to the diameter of droplets containing pathogens [53]. Studies have shown that a reduced rainfall lowers the relative humidity, decreasing the diameter of droplets containing pathogens, thereby facilitating their entry into the respiratory tract [53,54], and increasing the risk of tuberculosis.

In addition, the cattle distribution density and sheep distribution density are also important risk factors for tuberculosis. Based on the response curve for important variables, within a certain range, the risk of tuberculosis in cattle, buffaloes, sheep, and goats gradually increased with enhanced distribution densities of cattle and sheep. This tendency may be due to the increase in the scale of cattle and sheep breeding, which increases the probability of healthy and sick animals coming into contact, likely resulting in an epidemic of tuberculosis [55,56]. Therefore, in the process of livestock breeding, we should pay attention to the breeding density and the environmental health of the barn.

### 4.2. Potential High-Risk Distribution of Tuberculosis

The high-risk areas of tuberculosis among cattle, buffaloes, sheep, and goats were mainly distributed across South, North, East, and Northwest China. Among them, parts of Xinjiang, Qinghai, Ningxia Hui Autonomous Region, and Inner Mongolia are at a higher risk of tuberculosis due to their higher density of cattle and sheep distribution (Figure 3). Due to the economic development and industrialization in North and East China, the production of air pollutants (NO_2_ and O_3_) may increase, thereby increasing the risk of tuberculosis in cattle, buffaloes, sheep, and goats. In addition, the high-risk areas were extremely concentrated in Guangdong, Guangxi, and Hainan in South China, likely influenced by the high temperatures in these areas.

### 4.3. Recommendations for the Prevention and Control of Tuberculosis

Tuberculosis in cattle, buffaloes, sheep, and goats can seriously affect the healthy development of animal husbandry and even endanger human health. Studying the prevalence of tuberculosis can help identify specific communities or geographical areas with a heavy burden of tuberculosis. Moreover, for areas that have been plagued by tuberculosis, regular monitoring and updating of the prevalence data can evaluate the effectiveness of the existing interventions, and the localized approaches targeting communities can be adjusted based on the tuberculosis prevalence. Furthermore, studying the prevalence of tuberculosis in different species groups can also lead to the development of targeted prevention and control strategies. Regular monitoring, the enforcement of prevention protocols in the high-risk areas identified, and active cooperation with the country’s regular quarantine policy are necessary.

Our study used a MaxEnt model, which can not only help to identify “hot spots” of high prevalence of tuberculosis and clarify the priority areas for tuberculosis prevention and control but can also quantify the effects of different environmental variables (e.g., air pollutants, temperature, humidity, etc.) on the prevalence of tuberculosis, thereby identifying the environmental variables that have a significant impact on the prevalence of tuberculosis and providing a scientific basis for formulating targeted prevention and control measures. Finally, the input data of the model can be regularly updated, including new case reports and environmental variables, to maintain the timeliness and accuracy of the prediction results.

### 4.4. Limitations

In China, once livestock are found to be infected with tuberculosis, they are typically killed immediately to prevent further transmission. While collecting study data on animal populations, we did not include specific members of the MTBC. Hence, we could not determine the pathogens involved in the cases analyzed in this study. However, the MTBC refers to a group of species that are genetically extremely similar; thus, the prevalence of tuberculosis among animal species may not be significantly different. Furthermore, although we utilized pseudo-absence data to mitigate the sampling bias, the results may be influenced by other types of biases or missing information.

## 5. Conclusions

The high-risk areas of tuberculosis among cattle, buffaloes, sheep, and goats were mainly distributed across South, North, East, and Northwest China. Regarding the environmental variables, NO_2_, O_3_, mean temperature of the coldest quarter, and precipitation of the driest month significantly influenced the prevalence of tuberculosis. When the NO_2_ level was ≤50.8 μg/m^3^, the overall prevalence of tuberculosis showed a rapidly increasing trend. As the O_3_ level increased, the prevalence of tuberculosis initially decreased and then increased. Furthermore, the mean temperature of the coldest quarter was positively correlated, whereas the precipitation of the driest month was negatively correlated with the prevalence of tuberculosis. The findings of this study provide a valuable reference for the prevention and control of tuberculosis in cattle, buffaloes, sheep, and goats in China. In the future, the etiology of tuberculosis should be investigated to construct a more efficient risk prediction model.

## Figures and Tables

**Table 1:** Description of environmental variables.

Classification	Variable	Description	Unit	Included
Bioclimatic variables	Bio 1	Annual mean temperature	°C	N
	Bio 2	Mean diurnal range	°C	Y
	Bio 3	Isothermality	%	N
	Bio 4	Temperature seasonality	°C	N
	Bio 5	Max temperature of the warmest month	°C	Y
	Bio 6	Min temperature of the coldest month	°C	N
	Bio 7	Annual temperature range	°C	N
	Bio 8	Mean temperature of the wettest quarter	°C	N
	Bio 9	Mean temperature of the driest quarter	°C	N
	Bio 10	Mean temperature of the warmest quarter	°C	N
	Bio 11	Mean temperature of the coldest quarter	°C	Y
	Bio 12	Annual precipitation	mm	N
	Bio 13	Precipitation of the wettest month	mm	N
	Bio 14	Precipitation of the driest month	mm	Y
	Bio 15	Precipitation seasonality	/	Y
	Bio 16	Precipitation of the wettest quarter	mm	N
	Bio 17	Precipitation of the driest quarter	mm	N
	Bio 18	Precipitation of the warmest quarter	mm	N
	Bio 19	Precipitation of the coldest quarter	mm	N
Air pollutants	PM_2.5_	Level of particulate matter 2.5	μg/m^3^	N
	PM_10_	Level of particulate matter 10	μg/m^3^	Y
	SO_2_	Level of sulfur dioxide	μg/m^3^	Y
	NO_2_	Level of nitrogen dioxide	μg/m^3^	Y
	CO	Level of carbon monoxide	μg/m^3^	Y
	O_3_	Level of ozone	μg/m^3^	Y
Host distribution density	Cattle	Cattle distribution density	head/km^2^	Y
	Buffalo	Buffalo distribution density	head/km^2^	Y
	Sheep	Sheep distribution density	head/km^2^	Y
	Goat	Goat distribution density	head/km^2^	Y

N indicates that variables were not included in the model; Y represents that variables were included in the model. Max: maximum; Min: minimum.

**Table 2:** Percentage contribution of each variable.

Variables	Percent Contribution (%)
Level of nitrogen dioxide (NO_2_) Mean temperature of the coldest quarter (Bio 11) Cattle distribution density (Cattle)	34.1
	16.1
	9.6
Sheep distribution density (Sheep) Level of ozone (O_3_) Precipitation of the driest month (Bio 14) Precipitation seasonality (Bio 15)	7.1
	5.1
	4.9
	4.8
Max temperature of the warmest month (Bio 5) Goat distribution density (Goat)	3.9
	3.7
Level of sulfur dioxide (SO_2_)	2.6
Level of particulate matter 10 (PM_10_)	2.5
Isothermality (Bio 3)	2.4
Level of carbon monoxide (CO)	2.4
Buffalo distribution density (Buffalo)	0.8

Max: maximum.

## Data Availability

The data supporting this study are included within the article and Supplementary Materials. For further access to the data, please contact the corresponding authors.

## Supplementary Materials

The following supporting information can be downloaded at https://www.mdpi.com/article/10.3390/ani14243704/s1. Table S1: Specific information and sources of collected tuberculosis data for cattle, buffaloes, goats, and sheep. Table S2: Purified positional coordinates of the collected tuberculosis data for cattle, buffaloes, sheep, and goats.
