# Supplementary material for: Predicting Tuberculosis Risk in Cattle, Buffaloes, Sheep, and Goats in China Based on Air Pollutants and Meteorological Factors
Source: Animals (Basel). 2024 Dec 22;14(24):3704. doi: 10.3390/ani14243704 (PMC11672850; doi:10.3390/ani14243704)
Supplement: Supplementary file 1 [file animals-14-03704-s001.zip › Table S1.pdf]

Predicting Tuberculosis Risk in Cattle, Buffaloes, Sheep and Goats in China Based on Air Pollutants and Meteorological Factors

Le Xu, Suya Li, Hong Li, Haoju Pan, Shiyuan Li, Yingxue Yang, Yuqing Jiao, Feng Lan, Si Chen, Qiaoling Chen, Li Du, Churiga Man, Fengyang Wang, and Hongyan Gao*,

Hainan Key Laboratory of Tropical Animal Reproduction & Breeding and Epidemic Disease Research, School of Tropical Agriculture and Forestry, Hainan University, Haikou 570228, People's Republic of China.

Supplemental Files

Table S1 Detailed information of tuberculosis in cattle, buffaloes, sheep and goat: location of occurrence and reference.

No.	Species	Diseases	Location	Reference
1	Cow	Bovine tuberculosis	Liangzhou, Wuwei, Gansu	[1]
2	Cow	Bovine tuberculosis	Shanghai	[2]
3	Cow	Bovine tuberculosis	Lingwu, Ningxia	[3]
4	Cow	Bovine tuberculosis	Lanxi, Suihua, Heilongjiang	[4]
5	Cow	Bovine tuberculosis	Fangzhen, Daiyue, Taian, Shandong	[5]
6	Cattle	Bovine tuberculosis	Guizhou	[6]
7	Cow	Bovine tuberculosis	Zhaoyuan, Shandong	[7]
8	Cow	Bovine tuberculosis	Aksu, Xinjiang	[8]
9	Cow	Bovine tuberculosis	Fuhai, Altay, Xinjiang	[9]
10	Cow	Bovine tuberculosis	Jiayuguan, Gansu	[10]
11	Cow	Bovine tuberculosis	Huangzhong, Xining, Qinghai	[11]
12	Cow	Bovine	Huangyuan, Xining, Qinghai	[11]

		tuberculosis		
13	Cow	Bovine tuberculosis	Suzhou, Jiuquan, Gansu	[12]
14	Cow	Bovine tuberculosis	Urumqi, Xinjiang	[13]
15	Cow	Bovine tuberculosis	Liuzhou, Guangxi	[14]
16	Cow	Bovine tuberculosis	Longyang, Baoshan, Yunnan	[15]
17	Cow	Bovine tuberculosis	Hami, Xinjiang	[16]
18	Cow	Bovine tuberculosis	Nanning, Guangxi	[17]
19	Cow	Bovine tuberculosis	Changji, Xinjiang	[18]
20	Cow	Bovine tuberculosis	Chifeng, Inner Mongolia	[19]
21	Cow	Bovine tuberculosis	Chuankou, Minhe, Haidong, Qinghai	[20]
22	Cow	Bovine tuberculosis	Machangyuan, Minhe, Haidong, Qinghai	[20]
23	Cow	Bovine tuberculosis	Hetaozhuang, Minhe, Haidong, Qinghai	[20]
24	Cow	Bovine tuberculosis	Lierbao, Minhe, Haidong, Qinghai	[20]
25	Buffalo	Bovine tuberculosis	Nanning. Guangxi	[21]
26	Buffalo	Bovine tuberculosis	Wuxuan, Guangxi	[21]
27	Buffalo	Bovine tuberculosis	Laibin, Guangxi	[21]
28	Buffalo	Bovine tuberculosis	Wuming, Guangxi	[21]
29	Buffalo	Bovine tuberculosis	Hepu, Guangxi	[21]
30	Buffalo	Bovine tuberculosis	Qinzhou, Guangxi	[22]
31	Cow	Bovine tuberculosis	Yunnan	[23]
32	Cow	Bovine tuberculosis	Liuzhou, Guangxi	[24]
33	Cow	Bovine tuberculosis	Nanning, Guangxi	[24]

34	Cow	Bovine tuberculosis	Congming, Shanghai	[25]
35	Cow	Bovine tuberculosis	Banan, Chongqing	[26]
36	Cow	Bovine tuberculosis	Xinjiang	[27]
37	Cow	Bovine tuberculosis	Minhe, Haidong, Qinghai	[28]
38	Cow	Bovine tuberculosis	Tangshan, Hebei	[29]
39	Cow	Bovine tuberculosis	Qinhuangdao, Hebei	[29]
40	Cow	Bovine tuberculosis	Cangzhou, Hebei	[29]
41	Cow	Bovine tuberculosis	Shijiazhuang, Hebei	[29]
42	Cow	Bovine tuberculosis	Guizhou	[30]
43	Cow	Bovine tuberculosis	Luancheng, Shijiazhuang, Hebei	[31]
44	Cow	Bovine tuberculosis	Wujiaqu, Xinjiang	[32]
45	Cow	Bovine tuberculosis	Beipiao, Liaoning	[33]
46	Cow	Bovine tuberculosis	Minhe, Haidong, Qinghai	[34]
47	Cow	Bovine tuberculosis	Xining, Qinghai	[35]
48	Cow	Bovine tuberculosis	Huangyuan, Xining, Qinghai	[35]
49	Cow	Bovine tuberculosis	Huangzhong, Xining, Qinghai	[35]
50	Cow	Bovine tuberculosis	Datong, Xining, Qinghai	[35]
51	Cow	Bovine tuberculosis	Wudi, Binzhou, Shandong	[36]
52	Cow	Bovine tuberculosis	Jiangyan, Jiangsu	[37]
53	Cow	Bovine tuberculosis	Guangxi	[38]
54	Cow	Bovine tuberculosis	Xining, Qinghai	[39]
55	Cow	Bovine tuberculosis	Ruian, Zhejiang	[40]

56	Cow	Bovine tuberculosis	Qijiang, Chongqing	[41]
57	Cow	Bovine tuberculosis	Kunming, Yunnan	[42]
58	Cow	Bovine tuberculosis	Urumqi, Xinjiang	[43]
59	Cow	Bovine tuberculosis	Wenzhou, Zhejiang	[44]
60	Cow	Bovine tuberculosis	Dalian, Liaoning	[45]
61	Cow	Bovine tuberculosis	Liuyuan, Anxi, Jiuquan, Gansu	[46]
62	Cow	Bovine tuberculosis	Kashi, Xinjiang	[47]
63	Cow	Bovine tuberculosis	Arewusitang, Yining, Yili, Xinjiang	[48]
64	Cow	Bovine tuberculosis	Taixing, Jangsu	[48]
65	Cow	Bovine tuberculosis	Jilin	[49]
66	Cow	Bovine tuberculosis	Jinchuan, Jinchang, Gansu	[50]
67	Cow	Bovine tuberculosis	Qinghe, Tieling, Liaoning	[51]
68	Cow	Bovine tuberculosis	Eryuan, Dali, Yunnan	[52]
69	Cow	Bovine tuberculosis	Ningxia	[53]
70	Cow	Bovine tuberculosis	Jinfeng, Ningxia	[53]
71	Cow	Bovine tuberculosis	Jiuquan, Gansu	[54]
72	Cow	Bovine tuberculosis	Lanzhou, Gansu	[54]
73	Cow	Bovine tuberculosis	Gansu	[54]
74	Cow	Bovine tuberculosis	Changji, Xinjiang	[55]
75	Cow	Bovine tuberculosis	Eryuan, Dali, Yunnan	[56]
76	Cow	Bovine tuberculosis	Chongzuo, Guangxi	[57]
77	Cow	Bovine tuberculosis	Yancheng, Jiangzu	[58]

78	Cow	Bovine tuberculosis	Jiaxing, Zhejiang	[59]
79	Cow	Bovine tuberculosis	Xigu, Lanzhou, Gansu	[60]
80	Cow	Bovine tuberculosis	Guizhou	[61]
81	Cow	Bovine tuberculosis	Anning, Yunnan	[62]
82	Cow	Bovine tuberculosis	Gongjing, Zigong, Sichuan	[63]
83	Cattle	Bovine tuberculosis	Fuhai, Altay, Xinjiang	[64]
84	Cow	Bovine tuberculosis	Kaiyuan, Yunnan	[65]
85	Cow	Bovine tuberculosis	Urumqi, Xinjiang	[66]
86	Cow	Bovine tuberculosis	Changji, Xinjiang	[66]
87	Cow	Bovine tuberculosis	Hutubi, Xinjiang	[66]
88	Cow	Bovine tuberculosis	Yongjing, Linxia, Gansu	[67]
89	Cow	Bovine tuberculosis	Hongya, Meishan, Sichuan	[68]
90	Cow	Bovine tuberculosis	Dongpo, Meishan, Sichuan	[68]
91	Cow	Bovine tuberculosis	Anzhou, Mianyang, Sichuan	[68]
92	Cow	Bovine tuberculosis	Qionglai, Sichuan	[68]
93	Cow	Bovine tuberculosis	Jingyang, Deyang, Sichuan	[68]
94	Cow	Bovine tuberculosis	Zhaozhuang, Shandong	[69]
95	Cow	Bovine tuberculosis	Guangxi	[70]
96	Cow	Bovine tuberculosis	Liuzhou, Guangxi	[70]
97	Cow	Bovine tuberculosis	Yulin, Guangxi	[70]
98	Cow	Bovine tuberculosis	Xincheng, Laibin, Guangxi	[70]
99	Cow	Bovine tuberculosis	Wuxuan, Laibin, Guangxi	[70]

100	Cow	Bovine tuberculosis	Nanning, Guangxi	[70]
101	Cow	Bovine tuberculosis	Huanjiang, Hechi, Guangxi	[70]
102	Cow	Bovine tuberculosis	Shanglin, Nanning, Guangxi	[70]
103	Cow	Bovine tuberculosis	Wuxuan,Guangxi	[70]
104	Cow	Bovine tuberculosis	Yuzhou, Yulin, Guangxi	[70]
105	Cow	Bovine tuberculosis	Fumian, Yulin, Guangxi	[70]
106	Cow	Bovine tuberculosis	Xingye, Yulin, Guangxi	[70]
107	Cow	Bovine tuberculosis	Mengshan, Wuzhou, Guangxi	[70]
108	Cow	Bovine tuberculosis	Liuzhou, Guangxi	[70]
109	Cow	Bovine tuberculosis	Yulin, Guangxi	[70]
110	Cow	Bovine tuberculosis	Wuzhou, Guangxi	[70]
111	Cow	Bovine tuberculosis	Suining, Xuzhou, Jiangsu	[71]
112	Cow	Bovine tuberculosis	Shuangcheng, Heilongjiang	[72]
113	Cow	Bovine tuberculosis	Fengxian, Shanghai	[73]
114	Cow	Bovine tuberculosis	Taian, Shandong	[74]
115	Cattle	Bovine tuberculosis	Yongchuan, Chongqing	[75]
116	Cow	Bovine tuberculosis	Xincheng, Beiliu, Beiliu, Guangxi	[76]
117	Cow	Bovine tuberculosis	Mumian, Xilang, Beiliu, Guangxi	[76]
118	Cow	Bovine tuberculosis	Huancheng, Beiliu, Beiliu, Guangxi	[76]
119	Cow	Bovine tuberculosis	Tielu, Shanwei, Beiliu, Guangxi	[76]
120	Cow	Bovine tuberculosis	Doukou, Minyue, Beiliu, Guangxi	[76]
121	Cow	Bovine tuberculosis	Lanzhou, Gansu	[77]

122	Cow	Bovine tuberculosis	Tianshui, Gansu	[77]
123	Cow	Bovine tuberculosis	Wuwei, Gansu	[77]
124	Cow	Bovine tuberculosis	Zhangye, Gansu	[77]
125	Cow	Bovine tuberculosis	Pingliang, Gansu	[77]
126	Cow	Bovine tuberculosis	Dingxi, Gansu	[77]
127	Cow	Bovine tuberculosis	Longnan, Gansu	[77]
128	Cow	Bovine tuberculosis	Linxiazhou, Gansu	[77]
129	Cow	Bovine tuberculosis	Henan	[78]
130	Cattle	Bovine tuberculosis	Altay, Xinjiang	[79]
131	Cow	Bovine tuberculosis	Xifeng, Guiyang, Guizhou	[80]
132	Cow	Bovine tuberculosis	Guanshanhu, Guiyang, Guizhou	[80]
133	Cow	Bovine tuberculosis	Qingzhen, Guiyang, Guizhou	[80]
134	Cow	Bovine tuberculosis	Xiuwen, Guiyang, Guizhou	[80]
135	Cow	Bovine tuberculosis	Kaiyang, Guiyang, Guizhou	[80]
136	Cow	Bovine tuberculosis	Huayuan, Shihezi, Xinjiang	[81]
137	Cow	Bovine tuberculosis	Cuiping, Yibin, Sichuan	[82]
138	Cow	Bovine tuberculosis	Xinjiang	[83]
139	Cow	Bovine tuberculosis	Yulin, Shanxi	[84]
140	Cow	Bovine tuberculosis	Henan	[85]
141	Cow	Bovine tuberculosis	Miyun, Beijing	[86]
142	Cow	Bovine tuberculosis	Bazhou, Xinjiang	[87]
143	Cow	Bovine tuberculosis	Aksu, Xinjiang	[87]

144	Cow	Bovine tuberculosis	Urumqi, Xinjiang	[87]
145	Cow	Bovine tuberculosis	Changji, Xinjiang	[87]
146	Cow	Bovine tuberculosis	Hami, Xinjiang	[87]
147	Cow	Bovine tuberculosis	Fufeng, Baoji, Shanxi	[88]
148	Cow	Bovine tuberculosis	Jiangzhang, Fufeng, Baoji, Shanxi	[88]
149	Cow	Bovine tuberculosis	Xuzhou, Jiangsu	[89]
150	Cow	Bovine tuberculosis	Ruian, Zhejiang	[90]
151	Cow	Bovine tuberculosis	Laixi, Shandong	[91]
152	Cow	Bovine tuberculosis	Fengjie, Chongqing	[92]
153	Cow	Bovine tuberculosis	Linan, Hangzhou, Zhejiang	[93]
154	Cow	Bovine tuberculosis	Xiaoshan, Hangzhou, Zhejiang	[93]
155	Cow	Bovine tuberculosis	Yuhang, Hangzhou, Zhejiang	[93]
156	Cow	Bovine tuberculosis	Jianggan, Hangzhou, Zhejiang	[93]
157	Cow	Bovine tuberculosis	Gongye, Hangzhou, Zhejiang	[93]
158	Cow	Bovine tuberculosis	Jiande, Hangzhou, Zhejiang	[93]
159	Cow	Bovine tuberculosis	Fuyang, Hangzhou, Zhejiang	[93]
160	Cow	Bovine tuberculosis	Hangzhou, Zhejiang	[93]
161	Cow	Bovine tuberculosis	Hanzhong, Shanxi	[94]
162	Cow	Bovine tuberculosis	Aksu, Xinjiang	[95]
163	Cow	Bovine tuberculosis	Bazhou, Xinjiang	[95]
164	Cow	Bovine tuberculosis	Bozhou, Xinjiang	[95]
165	Cow	Bovine tuberculosis	Changji, Xinjiang	[95]

166	Cow	Bovine tuberculosis	Shihezi, Xinjiang	[95]
167	Cow	Bovine tuberculosis	Tacheng, Xinjiang	[95]
168	Cow	Bovine tuberculosis	Yili, Xinjiang	[95]
169	Cow	Bovine tuberculosis	Gansu	[96]
170	Cow	Bovine tuberculosis	Liuzhou, Guangxi	[97]
171	Cow	Bovine tuberculosis	Lasha, Tibet	[98]
172	Cow	Bovine tuberculosis	Shigatse, Tibet	[98]
173	Cow	Bovine tuberculosis	Ali, Tibet	[98]
174	Cow	Bovine tuberculosis	Naqu, Tibet	[98]
175	Cow	Bovine tuberculosis	Linzhi, Tibet	[98]
176	Cow	Bovine tuberculosis	Nangqian, Qinghai	[98]
177	Cow	Bovine tuberculosis	Qilian, Qinghai	[98]
178	Buffalo	Bovine tuberculosis	Nanning, Guangxi	[99]
179	Buffalo	Bovine tuberculosis	Wuxuan, Guangxi	[99]
180	Buffalo	Bovine tuberculosis	Laibin, Guangxi	[99]
181	Buffalo	Bovine tuberculosis	Hepu, Guangxi	[99]
182	Buffalo	Bovine tuberculosis	Wuming, Guangxi	[99]
183	Cow	Bovine tuberculosis	Shibing, Guizhou	[100]
184	Cow	Bovine tuberculosis	Shihezi, Xinjiang	[101]
185	Cow	Bovine tuberculosis	Yinchun, Jiangxi	[102]
186	Cow	Bovine tuberculosis	Jiyuan, Henan	[103]
187	Cow	Bovine tuberculosis	Shanghai	[104]

188	Cow	Bovine tuberculosis	Shihezi, Xinjiang	[105]
189	Cow	Bovine tuberculosis	Dalian, Liaoning	[106]
190	Cow	Bovine tuberculosis	Anshan, Liaoning	[106]
191	Cow	Bovine tuberculosis	Dandong, Liaoning	[106]
192	Cow	Bovine tuberculosis	Yingkou, Liaoning	[106]
193	Cow	Bovine tuberculosis	Fuxin, Liaoning	[106]
194	Cow	Bovine tuberculosis	Liaoyang, Liaoning	[106]
195	Cow	Bovine tuberculosis	Panjin, Liaoning	[106]
196	Cow	Bovine tuberculosis	Tieling, Liaoning	[106]
197	Cow	Bovine tuberculosis	Chaoyang, Liaoning	[106]
198	Cow	Bovine tuberculosis	Urumqi, Xinjiang	[107]
199	Cow	Bovine tuberculosis	Changji, Xinjiang	[107]
200	Cow	Bovine tuberculosis	Hutubi, Xinjiang	[107]
201	Cow	Bovine tuberculosis	Helan, Yinchuan, Ningxia	[108]
202	Cow	Bovine tuberculosis	Liwo, Qingzhen, Guizhou	[109]
203	Cow	Bovine tuberculosis	Linzhi, Tibet	[110]
204	Cow	Bovine tuberculosis	Yinchan, Ningxia	[111]
205	Cow	Bovine tuberculosis	Zhenhai, Ningbo, Zhejiang	[112]
206	Cow	Bovine tuberculosis	Yongjiu, Bayi, Linzhi, Tibet	[113]
207	Cow	Bovine tuberculosis	Jiangsu	[114
208	Cow	Bovine tuberculosis	Wuqing, Tianjing	[115]
209	Cow	Bovine tuberculosis	Ninghe, Tianjing	[115]

210	Cow	Bovine tuberculosis	Beichen, Tianjing	[115]
211	Cow	Bovine tuberculosis	Jixian, Tianjing	[115]
212	Cow	Bovine tuberculosis	Jinghai, Tianjing	[115]
213	Cow	Bovine tuberculosis	Baodi, Tianjing	[115]
214	Cow	Bovine tuberculosis	Hanjie, Tianjing	[115]
215	Cow	Bovine tuberculosis	Xichang, Sichuan	[116]
216	Cow	Bovine tuberculosis	Beijing	[117]
217	Cow	Bovine tuberculosis	Nanning, Guangxi	[118]
218	Cow	Bovine tuberculosis	Gabasongdu, Tongde, Hainan, Qinghai	[119]
219	Cow	Bovine tuberculosis	Bazhou, Xinjiang	[120]
220	Cow	Bovine tuberculosis	Yinchuan, Ningxia	[121]
221	Cow	Bovine tuberculosis	Yanping, Nanping, Fujian	[122]
222	Cow	Bovine tuberculosis	Hualong, Qinghai	[123]
223	Cow	Bovine tuberculosis	Cihansu, Qiabuqia, Gonghe, Qinghai	[124]
224	Cow	Bovine tuberculosis	Shangtamai, Qiabuqia, Gonghe, Qinghai	[124]
225	Cow	Bovine tuberculosis	Jialongtai, Qiabuqia, Gonghe, Qinghai	[124]
226	Cow	Bovine tuberculosis	Dongba, Qiabuqia, Gonghe, Qinghai	[124]
227	Cow	Bovine tuberculosis	Xixiangka, Qiabuqia, Gonghe, Qinghai	[124]
228	Cow	Bovine tuberculosis	Shangmei, Qiabuqia, Gonghe, Qinghai	[124]
229	Cow	Bovine tuberculosis	Xiamei, Qiabuqia, Gonghe, Qinghai	[124]
230	Cow	Bovine tuberculosis	Xitai, Qiabuqia, Gonghe, Qinghai	[124]
231	Cow	Bovine tuberculosis	Zhongfang, Huaihua, Hunan	[125]

232	Cow	Bovine tuberculosis	Datan, Qingshizui, Menyuan, Qinghai	[126]
233	Cow	Bovine tuberculosis	Miyun, Beijing	[127]
234	Cow	Bovine tuberculosis	Liuzhou, Guangxi	[128]
235	Cow	Bovine tuberculosis	Bole, Xinjiang	[129]
236	Cow	Bovine tuberculosis	Yili, Xinjiang	[130]
237	Cow	Bovine tuberculosis	Jinan, Liuan, Anhui	[131]
238	Cow	Bovine tuberculosis	Shanglin, Nanning, Guangxi	[132]
239	Cattle	Bovine tuberculosis	Urumqi, Xinjiang	[133]
240	Cow	Bovine tuberculosis	Hejing, Bazhou, Xinjiang	[134]
241	Cow	Bovine tuberculosis	Midong, Urumqi, Xinjiang	[135]
242	Cow	Bovine tuberculosis	Wuwei, Gansu	[136]
243	Cow	Bovine tuberculosis	Wuxi, Jiangsu	[137]
244	Cow	Bovine tuberculosis	Dulan, Haixi, Qinghai	[138]
245	Cow	Bovine tuberculosis	Xian, Shanxi	[139]
246	Cow	Bovine tuberculosis	Baoji, Shanxi	[139]
247	Cow	Bovine tuberculosis	Xianyang, Shanxi	[139]
248	Cow	Bovine tuberculosis	Weinan, Shanxi	[139]
249	Cow	Bovine tuberculosis	Sichuan	[140]
250	Cow	Bovine tuberculosis	Shanghai	[140]
251	Cow	Bovine tuberculosis	Lanzhou, Gansu	[141]
252	Cow	Bovine tuberculosis	Tianshui, Gansu	[141]
253	Cow	Bovine tuberculosis	Xining, Qinghai	[142]

254	Cow	Bovine tuberculosis	Haidong, Qinghai	[142]
255	Cow	Bovine tuberculosis	Haibei, Qinghai	[142]
256	Cow	Bovine tuberculosis	Yushu, Qinghai	[142]
257	Cow	Bovine tuberculosis	Xining, Qinghai	[143]
258	Cow	Bovine tuberculosis	Huangzhong, Qinghai	[143]
259	Cow	Bovine tuberculosis	Huangyuan, Qinghai	[143]
260	Cow	Bovine tuberculosis	Gonghe, Qinghai	[143]
261	Cow	Bovine tuberculosis	Xining, Qinghai	[144]
262	Cow	Bovine tuberculosis	Haidong, Qinghai	[144]
263	Cow	Bovine tuberculosis	Haibei, Qinghai	[144]
264	Cow	Bovine tuberculosis	Haixi, Qinghai	[144]
265	Cow	Bovine tuberculosis	Guoluo, Qinghai	[144]
266	Cow	Bovine tuberculosis	Qiabuqia, Gonghe, Hainan, Qinghai	[145]
267	Cow	Bovine tuberculosis	Mangla, Guinan, Hainan, Qinghai	[145]
268	Cow	Bovine tuberculosis	Wudang, Guiyang, Guizhou	[146]
269	Cattle	Bovine tuberculosis	Chuxiong, Chuxiong, Yunnan	[147]
270	Cattle	Bovine tuberculosis	Shuangbai, Chuxiong, Yunnan	[147]
271	Cattle	Bovine tuberculosis	Yaoan, Chuxiong, Yunnan	[147]
272	Cattle	Bovine tuberculosis	Yuanmou, Chuxiong, Yunnan	[147]
273	Cow	Bovine tuberculosis	Tengchong, Yunnan	[148]
274	Cow	Bovine tuberculosis	Mudan, Heze, Shandong	[149]
275	Cow	Bovine tuberculosis	Zhenqin, Chenduo, Yushu, Qinghai	[150]

276	Cow	Bovine tuberculosis	Wuxi, Jiangsu	[151]
277	Cow	Bovine tuberculosis	Xiajinbatai, Beishan, Menyuan, Haibei, Qinghai	[152]
278	Cow	Bovine tuberculosis	Beishangen, Beishan, Menyuan, Haibei, Qinghai	[152]
279	Cow	Bovine tuberculosis	Changning, Baoshan, Yunnan	[153]
280	Cow	Bovine tuberculosis	Midu, Dali, Yunnan	[154]
281	Cow	Bovine tuberculosis	Linxia, Gansu	[155]
282	Cow	Bovine tuberculosis	Shangluowa, Mangla, Guinan, Hainan, Qinghai	[156]
283	Cow	Bovine tuberculosis	Chana, Shagou, Guinan, Hainan, Qinghai	[156]
284	Cattle	Bovine tuberculosis	Wensu, Aksu, Xinjiang	[157]
285	Cow	Bovine tuberculosis	Chenbaerhu, Hulunbeier, Inner Mongolia	[158]
286	Cow	Bovine tuberculosis	Lujiashan, Jinbian, Yulin, Shanxi	[159]
287	Cow	Bovine tuberculosis	Haicetan, Jinbian, Yulin, Shanxi	[159]
288	Cow	Bovine tuberculosis	Miaopan, Jinbian, Yulin, Shanxi	[159]
289	Cow	Bovine tuberculosis	Menyuan, Haibei, Qinghai	[160]
290	Cow	Bovine tuberculosis	Qilian, Haibei, Qinghai	[160]
291	Cow	Bovine tuberculosis	Haiyan, Haibei, Qinghai	[160]
292	Cow	Bovine tuberculosis	Yangcheng, Jinyang, Shanxi	[161]
293	Cow	Bovine tuberculosis	Saishenke, Wulan, Haixi, Qinghai	[162]
294	Cow	Bovine tuberculosis	Huangyan, Taizhou, Zhejiang	[163]
295	Cow	Bovine tuberculosis	Hangzhou, Zhejiang	[164]
296	Cow	Bovine tuberculosis	Datong, Xining, Qinghai	[165]
297	Cow	Bovine tuberculosis	Ganggou, Ledu, Haidong, Qinghai	[166]

298	Cow	Bovine tuberculosis	Hunan	[167]
299	Cow	Bovine tuberculosis	Minhe, Haidong, Qinghai	[168]
300	Cow	Bovine tuberculosis	Huangnan, Qinghai	[169]
301	Cow	Bovine tuberculosis	Yanping, Nanping, Fujian	[170]
302	Cow	Bovine tuberculosis	Xinglong, Dongan, Mudanjiang, Heilongjiang	[171]
303	Cow	Bovine tuberculosis	Dongcun, Dongan, Mudanjiang, Heilongjiang	[171]
304	Cow	Bovine tuberculosis	Tieling, Yangming, Mudanjiang, Heilongjiang	[171]
305	Cow	Bovine tuberculosis	Hami, Xinjiang	[172]
306	Cow	Bovine tuberculosis	Ningbo, Zhejiang	[173]
307	Cow	Bovine tuberculosis	Altay, Xinjiang	[174]
308	Cow	Bovine tuberculosis	Jiangbian, Yulin, Shanxi	[175]
309	Cow	Bovine tuberculosis	Xinghua, Jiangsu	[176]
310	Cow	Bovine tuberculosis	Anding, Dingxi, Gansu	[177]
311	Cow	Bovine tuberculosis	Shache, Xinjiang	[178]
312	Cow	Bovine tuberculosis	Huangzhou, Huanggang, Hubei	[179]
313	Cow	Bovine tuberculosis	Guangzhou, Guangdong	[180]
314	Cow	Bovine tuberculosis	Anyang, Henan	[181]
315	Cow	Bovine tuberculosis	Linxian, Anyang, Henan	[181]
316	Cow	Bovine tuberculosis	Shijiagou, Dongjiao, Wenfeng, Anyang, Henan	[181]
317	Cow	Bovine tuberculosis	Longan, Anyang, Henan	[181]
318	Cow	Bovine tuberculosis	Beiguan, Anyang, Henan	[181]
319	Cow	Bovine tuberculosis	Wenfeng, Anyang, Henan	[181]

320	Cow	Bovine tuberculosis	Shaoguan, Guangdong	[182]
321	Cow	Bovine tuberculosis	Qingzhen, Guizhou	[183]
322	Cow	Bovine tuberculosis	Litong, Wuzhong, Ningxia	[184]
323	Cow	Bovine tuberculosis	Tianshan, Xinjiang	[185]
324	Cow	Bovine tuberculosis	Tongwei, Dingxi, Gansu	[186]
325	Cow	Bovine tuberculosis	Lintao, Dingxi, Gansu	[186]
326	Cow	Bovine tuberculosis	Weifang, Shandong	[187]
327	Cow	Bovine tuberculosis	Hongya, Meishan, Sichuan	[188]
328	Cow	Bovine tuberculosis	Wuzhou, Guangxi	[189]
329	Cow	Bovine tuberculosis	Qumalai, Yushu, Qinghai	[190]
330	Cow	Bovine tuberculosis	Jimsar, Xinjiang	[191]
331	Cow	Bovine tuberculosis	Zijiao, Shinian, Wenling, Zhejiang	[192]
332	Sheep	Sheep tuberculosis	Guilin, Guangxi	[193]
333	Sheep	Sheep tuberculosis	Qingtongxia, Ningxia	[194]
334	Goat	Goat tuberculosis	Guizhou	[195]

References

1. Wang M.K., Ren J. Treatment of a positive cow tuberculosis quarantine. *China Cattle Science* **2020** *46(02)*, 95-96. **(in Chinese)**
2. Lu J., Sun Q.Y., Xia L.M., Zhao H.J., Wang Q.Z., Wang G.L., et al. Emergency epidemiological investigation of a quarantine positive cow tuberculosis. *China Animal Health Inspection*. **2015.** *32(08)*, 4-7. **(in Chinese)**
3. Li X.M., He Y.X., Sha Y.B., Wei Z.F. Management and reflection on a case of tuberculosis in dairy cows. *Journal of Veterinary Sciences* **2004**, *(03)*, 58-59. (**in Chinese**)
4. Zhang H. A report of double positive detection of tuberculosis and brucellosis in sold dairy cows. *Technical Advisor for Animal Husbandry*. **2010** *(01)*, 138-139. (**in Chinese**)
5. Zhu Z.T., Liu S.J., Xue Q.Y., Zhang G.Q., Zhao Y. Treatment and Thinking of a Case of Tuberculosis Cow. *Shangdong Journal of Animal Science and Veterinary Medicine*. **2009**, *30(11)*, 43. **(in Chinese)**
6. Mo X.H., Liao F., Bao T.T, Ynag X.F., Zhao X.M. Diagnosis and experience of a case of beef cattle tuberculosis infection. *Chinese Abstracts of Animal Husbandry and Veterinary Medicine*. **2018** 34(01), 184. **(in Chinese)**

7. Teng Y.J., Zheng L.L. Diagnosis of a suspected cow intestinal tuberculosis. *Journal of Zhengzhou College of Animal Husbandry Engineering*. **2008**, *28(04)*, 27-28. **(in Chinese)**
8. Zhang Y.C. Epidemiological Investigation of Tuberculosis and Brucellosis in Dairy Cows of the First Agricultural Division. *XINJIANG XUMUYE* **2009** *(06)*, 51. **(in Chinese)**
9. Pu X.Z., Gu H.Y., Li Y.M., Li W., Hu J.W. The quarantine measures of ' two diseases ' of 181 regiment dairy cows. *Xinjiang Farm Research Science an Technology*. **2006**, *(06)*, 42. **(in Chinese)**
10. He G.Y., Zhang Y.Q. Census and monitoring purification measures of ' two diseases ' of dairy cows in Jiayuguan City. *Journal of Animal Science and Veterinary Medicine.* **2018**, *37(01)*, 40-41,43 **(in Chinese)**.
11. Li X.Y., Ma R.L., Wang Y.P., Wang S.X., Fu Y.J. Investigation on Tuberculosis Infection of Dairy Cows in Huangzhong and Huangyuan Counties of Qinghai. *Chinese Journal of Animal Health Inspection*. **2014**, *31(07)*, 73-75. **(in Chinese)**
12. Wang J.Y. Analysis and Countermeasures of ' Two Diseases ' Quarantine of Dairy Cows in Suzhou District. *Agricultural Science-Technology and Information* **2007**, *(16)*, 69-70. **(in Chinese)**
13. Wang L.H., Xu M., Xia J., Ma W.P., Wang W.X., Sun G.L. Discussion and experience of different culling methods for ' two diseases ' quarantine positive cows. *XINJIANG XUMUYE*. **2011,** *(03)*, 49-51. **(in Chinese)**
14. Bian D.Y., Zhu X., Deng H.Y., Lu J. Discussion on Legal Issues of Cow Two Diseases Treatment in Industrial Development Zone. *The Chinese Livestock and Poultry Breeding*. **2011**, *7(09)*, 23-24. (**in Chinese**)
15. Yang H.F. Thoughts on the monitoring of ' two diseases ' in dairy cows in Longyang District. *Yunnan Journal of Animal Science and Veterinary Medicine*. **2007**. *(z1),* 37-38. (**in Chinese**)
16. Chen J.H., Duan J.G., Abudureyimu S., Zhang Z.X., Yunvsi A, Ma C.J. The application of two detection methods in bovine tuberculosis quarantine. *The Chinese Livestock and Poultry Breeding*. **2020**, *16(12)*, 59-60. **(in Chinese)**
17. Zhang L. 2020. Comparison of two detection methods for bovine tuberculosis. nongjia zhifu guwen. (10), 122. **(in Chinese)**
18. Fan B.J. Bayesian evaluation and application of diagnostic methods for bovine tuberculosis. *Xinjiang Agricultural University*. **2020 (in Chinese)**
19. Shung G., Su L.D., Hasituya., Si Q.F. Problems and countermeasures in the monitoring and purification of ' two diseases ' in dairy cows. *Veterinary Orientation*. **2020**, *(03)*, 53,70. **(in Chinese)**
20. Li S.F., Li Z.H. Zhong W.D. Investigation on the prevention and control effect of ' two diseases ' of dairy cows in Minhe County. *Chinese Qighai Journal of Animal and Veterinary Sciences*. **2017**,*47(03)*, 33-34. **(in Chinese)**
21. Xie Z.Q., Xie Z.X., Liu J.B., Pang Y.S., Deng X.W., Xie L.J., et al. Comparison of four methods for detection of tuberculosis in Guangxi dairy buffalo. *China Animal Health Inspection*. **2010**, *27(11)*, 52-55. **(in Chinese)**
22. Lao Y.R., Huang M., Cao P.Y., Li C.Y. Epidemiological investigation of ' two diseases ' in some dairy buffalo farms in Qinzhou area of Guangxi. *Livestock and Poultry Industry*. **2015**, *(08)*, 65-66. **(in Chinese)**
23. Wang X.Q., Li F.X., Zhao W.H., Wang J.P., Yang S.B. Study on the detection of bovine tuberculosis by two in vivo diagnostic methods combined with etiological diagnosis. *China Animal Health.* **2012**, *14(04)*, 10-14,94. **(in Chinese)**
24. Xu X.K., Huang X.W., Lan J., Huang S.B., Xiong Y., Liu Q., et al. Comparison of two detection

methods of cow tuberculosis. *Animal Husbandry and Veterinary Medicine*. **2010**, *42(02)*, 29-33. **(in Chinese)**

25. Ye H.P., Qin J., Shi B., Yuan J.J., Fu G.X., Shi C.P., et al. Monitoring on regional purification of ' two diseases ' of dairy cows in Chongming District of Shanghai. *Chinese Journal of Veterinary Medicine*. **2021**, *57(03)*, 108-111. **(in Chinese)**
26. Yang Y. The prevention and control effect of ' two diseases ' of cattle and sheep in Banan District. *Animal Indusry and Environment.* **2021**, *(8)*, 58. **(in Chinese)**
27. Shi Q., Yuan L.G., Pu J.W., Sha L., LiuW. Comparison of the coincidence rate between the two methods of tuberculosis detection and PPD detection in dairy cows. *China Animal Health Inspection.* **2018**, *35(10),* 87-89. **(in Chinese)**
28. Li S.F., Zhong W.D., Li Z.H. Study on the detection of "two diseases" in dairy cows "out of the house into the garden" in Minhe county. *Chinese Qighai Journal of Animal and Veterinary Sciences*. **2016**, *46(01)*, 23-24. **(in Chinese)**
29. Wang L.X., Li A.H., Wang H.J., Liu C.M., Liu T,J., Li C., et al. Comparative analysis of two methods for detecting tuberculosis in dairy cows. *China Animal Health*. **2015**, 66-68. **(in Chinese)**
30. Yang L., Liu F.Q., Song D.S., Sun Q.Y., Tian M.L., Wu W.H., et al. Comparative test of two detection methods of cow tuberculosis. *Guizhou Journal of Animal Husbandry and Veterinary Medicine*. **2014**, *38(04)*, 16-18. (in Chinese)
31. Liu Y., Zhang T., Cheng L.H., Bian Q.Y., Shi X.Y., ZUO Y.Z., et al. Monitoring and analysis of ' two diseases ' of dairy cows in Luancheng District of Shijiazhuang City in 2019. *BeiFang MuYe*. **2019**, *(23)*, 19-21. **(in Chinese)**
32. Ma C.B., Liu X.P., Chen W.W., Cui L., Wang Z.S. Zhang Z.R. Retrospective analysis of monitoring data of ' two diseases ' of cattle in different breeding modes. *China Cattle Science.* **2015**,*41(05)*, 53-55. **(in Chinese)**
33. Zhang S. Detection and purification of ' two diseases ' of dairy cows in Beipiao area of Liaoning Province. *China Animal Health.* **2018**, *20(05)*, 11-12. **(in Chinese)**
34. Hu Y.J. Investigation and Research on the Monitoring of ' Two Diseases ' of Dairy Cows in Minhe County. *China Cattle Science.* **2010**, *36(04)*, 85,89. **(in Chinese)**
35. Chen Y.Z., Zhang C.T., Zhen S.P. Investigation on the prevention and control of ' two diseases ' of dairy cows in Xining area. S*hanghai Journal of Animal Husbandry and Veterinary Medicine.* **2014**, *(01)*, 74-75. **(in Chinese)**
36. Wang X., Meng Y., Yang X.L., Chen J.M. Problems and Countermeasures in the positive treatment of ' two diseases ' in dairy cows. *Today Animal Husbandry and Veterinary Medicine*. **2010**, *(09)*, 64. **(in Chinese)**
37. Ji Y., Xu Z.Q., Wang H., Zhang H.P. Investigation on prevention and control of ' two diseases ' in dairy cows in Jiangyan City. *Shanghai Journal of Animal Husbandry and Veterinary Medicine.* **2009**, *(04)*, 83. **(in Chinese)**
38. Zou L.B., Guo J.G., Huang X., Li H.M., Liu Q. Two kinds of bovine tuberculosis detection methods for the detection of dairy cows in Guangxi. *China Animal Health Inspection.* **2007**, *(08)*, 24, 37-38.**(in Chinese)**
39. Ji S.H., Qi Y.X. Monitoring and quarantine of ' two diseases ' of dairy cows in Xining City. *Journal of Animal Science and Veterinary Medicine*. **2005**, *(02)*, 34-35. **(in Chinese)**
40. Lu R.Y., Peng N.M. The main measures to prevent and control the ' two diseases ' of dairy cows in Rui 'an City. *Zhejiang Journal of Animal Science and VerterInary Medicine.* **2005**, *(01),* 27. **(in**

Chinese)
41. Liu D.H., Feng L. Analysis and Countermeasures of Two Diseases Monitoring in Dairy Cows. *Stockbreeding Market.* **2004**, *(08)*, 39-40. **(in Chinese)**
42. Wei M., Li N., He Y.F., Wan B.Y. Monitoring and Thinking of Dairy Cows ' Two Diseases '. *China Animal Health Inspection*. **2004**, *(09)*, 31. **(in Chinese)**
43. Xu M., Chen X.H., Li J.L., Chen B., Zhang J., Huang X.W. Analysis and Countermeasures of ' Two Diseases ' Quarantine of Cows in Urumqi. *China Animal Health Inspection.* **2003**, *(01)*, 14-15. **(in Chinese)**
44. Xiang Y.R. The main purification measures of ' two diseases ' of dairy cows in Wenzhou City. *Zhejiang Journal Animal Science and Veterinary Medicine.* **2016,** *41(03)*, 15-15. **(in Chinese)**
45. Jin F.J., Fan Y. Review and Discussion on Purification of ' Two Diseases ' in Dairy Cows. *Chinese Journal of Animal Husbandry & Veterinary Medicine*. **2010**, *(01)*, 34-35. **(in Chinese)**
46. Li Z.S. Persistent quarantine to purify the two diseases of dairy cows. *Journal of Animal Science and Veterinary Medicine.* **2009**, *28(01)*, 92-93. **(in Chinese)**
47. Yang Q., Xie F. Investigation and Analysis of the Epidemic Situation of Two Diseases of Dairy Cows in Kashi Area and Discussion on Prevention and Control Measures. *China Animal Health Inspection.* **2008**, *25(09),* 36-37. **(in Chinese)**
48. Xiaokaiti N. Study on the influence of different quarantine methods on the positive rate of tuberculosis in dairy cows. *nongjia zhifu guwen*. **2020**. *(12)*, 111. **(in Chinese)**
49. Wang N., Wang Z.Y., Jiang X.L., Yu Q.L., Shi C.J. Comparative study on the application of four different detection methods in the diagnosis of tuberculosis in dairy cows. *Jilin Animal Science and Veterinary Medicine*. **2012**, *33(11)*, 19-20. **(in Chinese)**
50. Zhang H.F., QinJ., Zhao C.H., Zhao Y.H., Li X.X. Jinchuan District Dairy Cow Tuberculosis Detection Technology Test. *Gansu Animal and Veterinary Sciences.* **2020**, *50(03)*, 71-73. **(in Chinese)**
51. Fu L.G., Wang W., Zhao Y.C. Comprehensive measures for quarantine and monitoring of brucellosis and tuberculosis in dairy cows. *Helongjiang Animal Science and Veterinary Medicine.* **2004**, *(05)*, 77. **(in Chinese)**
52. Du P.W. Report on the detection of brucellosis and tuberculosis in dairy cows in Eryuan County. *Chinese Abstracts of Animal Husbandry and Veterinary Medicine.* **2017**, *33(09)*, 125. **(in Chinese)**
53. Tan Q., Gao A.X. Suggestions on detection and prevention of brucellosis and tuberculosis in dairy cows. *Contemporary Animal Husbandry.* **2015,** *(21)*, 15-16. **(in Chinese)**
54. Li Y.Z. Monitoring situation and prevention and control countermeasures of cow tuberculosis in Gansu Province. *Journal of Animal Science and Veterinary Medicine*. **2015,** *34(05)*, 113-114. **(in Chinese)**
55. Fan B.J., Zhang F., Xiao K.T., Amanguli., Huang J. Two immunological diagnostic methods of bovine tuberculosis were evaluated by Bayesian model. *China Animal Health Inspection.* **2020**. *37(06)*, 86-89. **(in Chinese)**
56. He R.H., Du P.W., Zhang G.F. Serological Investigation of Brucellosis and Tuberculosis in Dairy Cows in Eryuan County. *Yunnan Journal of Animal Science and Veterinary Medicine.* **2009,** *(03)*, 17-18. **(in Chinese)**
57. Huang G.M., Wei C.K. Quarantine and purification of bovine tuberculosis and brucellosis in Chongzuo City. *Guangxi Journal of Animal Huabandry & Veterinary Medicine.* **2006**, *(04)*, 168-170. **(in Chinese)**

58. Yi Q.Y., Bian H.C. Detection and analysis of tuberculosis and brucellosis in dairy cows in Yancheng area. *China Animal Health Inspection.* **2005**, *(11)*, 27. **(in Chinese)**
59. Jiang H., Zhang J.M., Zhang S.D. Purification of tuberculosis in large-scale dairy farms. *Jilin Animal Husbandry and Veterinary Medicine*. **2004.** *(02),* 47. **(in Chinese)**
60. Ren Y. Investigation on tuberculosis infection of dairy cows in Xigu District of Lanzhou City. *China Animal Health Inspection*. **2003**, *(12)*, 41. **(in Chinese)**
61. Ye L. Investigation on Infection of Tuberculosis and Brucellosisin Dairy Cows in Guizhou Province and Establishment ofMilk Safety Evaluation System. *Guizhou University*. **2019. (in Chinese)**
62. Li J.H., Liu Q., Zhou Y.P., Zhang Q.P., Zhang L.K., Shi K.P., et al. Comparative experiment of cow tuberculosis intradermal allergy and γ-interferon method detection. *The Chinese Livestock and Poultry Breeding.* **2018,** *14(04)*, 128-129. **(in Chinese)**
63. Ran X.F. Epidemiological investigation report of brucellosis and tuberculosis in dairy cows in Gongjing District of Zigong City. *Sichuan Agricultural University*. **2018. (in Chinese)**
64. Ye G.X., Zeng X.W., Yang F.X., Luo W.Y., Liu Y.H. Our division of a large-scale cattle farm brucellosis, tuberculosis quarantine and health analysis. *The Chinese Livestock and Poultry Breeding.* **2007,** *(08)*, 83-84. **(in Chinese)**
65. He J., Pu J.H., Lu Y.F., Fan X., Pu G.Y. Investigation and Prevention of Dairy Cow Tuberculosis in Kaiyuan City. Thesis Collection of the Sixth Academic Annual Conference of Yunnan Association for Science and Technology and Red River Basin Development Forum, *Yunnan Honghe*. **2016**, *pp.* 1-2. **(in Chinese)**
66. Salitanati J. Investigation of bovine tuberculosis infection on milk around Urumqi. *Xinjiang Agricultural University*. **2013.** **(in Chinese)**
67. Wu Z.C. Dairy cow tuberculosis quarantine and pathogen identification test in Yongjing County. *Gansu Animal Husbandry and Veterinary.* **2001,** *(01)*, 10-11. **(in Chinese)**
68. Deng Y.Q., Yang A.G., Guo L., Hou W., Chen D., Wen H., et al. Purification demonstration of tuberculosis and brucellosis in Sichuan dairy cattle. *China Animal Health Inspection*. **2014,** *31(08)*, 55-57. **(in Chinese)**
69. Zhang R., Chen P., Xu R,L., Li Y.Y. Epidemiological investigation of brucellosis and tuberculosis in cattle and sheep in Shizhong District of Zaozhuang City. *Shandong Journal of Animal Science and Veterinary Medicine*. **2015,** *36(09)*, 62-63. **(in Chinese)**
70. Gan H.X., Surveillance and Control of Dairy Cow Tuberculosis in Guangxi. *Guangxi University*. **2008. (in Chinese)**
71. Wang B.Q. Epidemiological Investigation of Brucellosis andTuberculosis in Dairy Cows and Demonstration ofComprehensive Prevention and Control Technology inSuining County of Jiangsu Province in the Period from2018 to 2020. *Yangzhou University*. **2021. (in Chinese)**
72. Song D.H., Han S., Zhang B.Q. Investigation on quarantine and purification of tuberculosis in suburban dairy farms in Shuangcheng City. *China Animal Health Inspection.* **2011,** *28(04)*, 60-61. **(in Chinese)**
73. Zhai H., Wang G.X., Shi H.H., Hong Y.C., Yang T., Gu X. Discussion on PPD retest results of dairy cows in Fengxian area. *Shanghai Journal of Animal Husbandry and Veterinary Medicine.* **2013**, *(04)*, 59. (in Chinese)
74. Chen H., Zheng X.H. Investigation and Analysis of Brucellosis and Tuberculosis Prevention and Control in Tai 'an City. *Shandong Journal of Animal Science and Veterinary Medicine*. **2009,** *30(01)*, 36-37. **(in Chinese)**

75. Wen F.Y. Discussion and experience of different culling methods for ' two diseases ' quarantine positive cows in dairy cows. *Nongjia Zhifu Guwen*. **2020,** *(22)*, 131. **(in Chinese)**
76. Cai M.Y. Epidemiological investigation of bovine tuberculosis in Beiliu City (County) of Guangxi. *Animals Breeding and Feed.* **2023**, *22(01)*, 77-81. **(in Chinese)**
77. Che X.J., Han Q.Y., Luo L.N., Wang L., Qing L., Meng LM., et al. Investigation and analysis of tuberculosis infection in dairy cows in Gansu Province from 2016 to 2020. *Shandong Journal of Animal Science and Veterinary Medicine*. **2022,** *43(07)*, 6-9. **(in Chinese)**
78. Zhao S.J., Ni B., Sheng M. Investigation on bovine tuberculosis infection in Henan Province in 2020. *Animals Breeding and Feed.* 2022, *21(02)*, 13-15. **(in Chinese)**
79. Luo P.F., Wang J.Q.. Epidemiological investigation of bovine tuberculosis in Altay region of Xinjiang. *Chinese Journal of Veterinary Medicine.* **2020,** *56(04)*, 83-86. **(in Chinese)**
80. Xu C,Z., Zhang H., Lin L.Z., Ynag Q.X., Yang Y., Liu Y., et al. Investigation and Analysis of Tuberculosis Infection in Cows in Guiyang City. *Modern Animal Husbandry.* **2021,** *(02)*, 17-18. **(in Chinese)**
81. Wang Z.S., Chen Q.B., Zhao Y.M. Detection report of cow tuberculosis in Huayuan town. *Veterinary Orientation.* **2020**, *(18)*, 214. **(in Chinese)**
82. Pan Y.L. Chen M., Wang Y.W. Application of three detection methods in the quarantine of bovine tuberculosis in Cuiping District. *China Animal Health.* **2023**, *25(02)*, 124-126. **(in Chinese)**
83. Lin K., Tian L.L., Chang T.N., Wen J.X., Fan W.X. Comprehensive diagnosis and pathogen isolation and identification of bovine tuberculosis in a cattle farm in Xinjiang. *Chinese Journal of Zoonoses*. **2019**. *35(12),* 1150-1153. **(in Chinese)**
84. Wu Y., Sheng J., Wu Q.M., Jia F.J., Liu Y.P., Cao C.L., et al. Detection and analysis of tuberculosis and brucellosis in dairy cows in a county of Yulin City. *Progress in Veterinary Medicine.* **2015,** *36(06)*, 186-188. **(in Chinese)**
85. Zhao S.J., Ni B., Sheng M. Investigation on bovine tuberculosis infection in Henan Province in 2020. *Animals Breeding and Feed*. **2022,** *21(02)*, 13-15. (in Chinese)
86. Zhang Q.L., Shen G.N., Zheng X.Y., Zhou D.G., Zhang W., Liu H.Y., et al. Field epidemiological investigation of tuberculosis in a dairy farm in Miyun District of Beijing. *China Animal Health Inspection.* **2019,** *36(07)*, 20-23. **(in Chinese)**
87. Li L., Xiaokaiti A., Amanguli, Li S., Fan B.J. Reyihanguli. Analysis of tuberculosis surveillance results of dairy cows in Xinjiang in 2018. *XINJIANG XUMUYE.* **2019**, *34(04)*, 33-35. **(in Chinese)**
88. Lv B., Xian J.L., Hai X.Z., Zhu W.C., Li Y.F., Zang W.Q. Analysis of tuberculosis detection results of dairy cows in Fufeng County.*Progress in Veterinary Medicine.* **2007,** *(07)*, 113-114. **(in Chinese)**
89. Gao A.P., Han F.W., Qi L., Duan S.H. Monitoring results and analysis of brucellosis and tuberculosis in dairy cows in Xuzhou area of Jiangsu Province. *Animal Husbandry and Veterinary Medicine.* **2004,** *(05)*, 31-32. **(in Chinese)**
90. Jin D.C., Wang Y.C., Hu Y.F., Jin J.J., Wang Z.Y. Investigation and epidemic trend analysis of tuberculosis infection in dairy cows. *China Animal Husbandry and Veterinary Medicine.* **2011,** *38(12)*, 200-202. **(in Chinese)**
91. Liu A.L., Ma Q.X., Han X., Shen C.J., Kang J.L., Zhang Y., et al. Analysis of prevalence and risk factors of tuberculosis in dairy cows in Laixi City, Shandong Province. *China Animal Health Inspection.* **2019,** *36(02)*, 17-21,41. **(in Chinese)**
92. Li Q.Y., Zhang D.Z., Cao L.T., Xiong Z.L., Wu B.Q., Zuo F.Y. Investigation on Tuberculosis Brucellosis of Beef Cattle in Three Gorges Reservoir Area of Chongqing. *Chinese Journal of*

Veterinary Medicine. **2016,** *52(04)*, 3-5. (in Chinese)
93. Xie Z., Li X.L., Fang W. H., Ying X.F., Tang L.P., Investigation on the prevalence of bovine tuberculosis in Hangzhou. *Acta Agriculturae Zhejiangensis.* **2007**, *(04)*, 310-313. **(in Chinese)**
94. Wang Y.X., Yu S.K., Zhai J.J. Investigation on Dairy Cow Tuberculosis in Hanzhong Area. *Progress in Veterinary Medicine.* **2007,** *(07)*, 111-113. **(in Chinese)**
95. Li D., Sheng Z.J., Liu J., Peng X.L., Qiao H.W., Chen D.K. PPD monitoring and analysis of bovine tuberculosis in some areas of Xinjiang. *Progress in Veterinary Medicine*. **2006,** *(04)*, 99-102. **(in Chinese)**
96. Yu Q.L,. Analysis of monitoring results and prevention and control suggestions of dairy cow tuberculosis in Gansu Province from 2011 to 2016. *China Dairy Cattle.* **2018**, *(01)*, 27-29. **(in Chinese)**
97. Yan S.G, Wei Z.J.,Huang X.W. Epidemiologic surveillance and analysis of tuberculosis in dairy cattle in Liuzhou City, China. *China Dairy Cattle* **2014**, *(14)*:21-23. **(in Chinese)**
98. Han Z.Q., Gao J.F., Yao R.S., Zhang K.R., Liu M.Y, Zhang D, et al. Serological detection report of yak tuberculosis in some areas of Qinghai-Tibet Plateau. *China Dairy Cattle.* **2012,** *(21)*, 34-35 **(in Chinese)**.
99. Xie Z.Q., Xie Z.X., Liu J.B., Pang Y.S., Deng X.W., Xie L.J., et al. Comparison of three detection methods for milk buffalo tuberculosis in Guangxi. *Southwest China Journal of Agricultural Sciences.* **2011**, *24(03)*, 1122-1125. **(in Chinese)**
100. Wu W.H., Yang L., Jia H.J., Yang M.S., Wen C.Z., Wu W.X., et al. Application of colloidal gold test strip diagnostic method to detect bovine tuberculosis test. *Guizhou Agricultural Sciences.* **2008,** *36(02)*, 121-122. **(in Chinese)**
101. Wang H.W., Li X.P., Li J., Shi W.J., Li L.J., Zhang X.E., et al. Comparative Study on Detection Methods of Bovine Tuberculosis in Shihezi region of Xinjiang. *China Dairy Cattle.* **2020,** *(07)*, 33-35. **(in Chinese)**
102. Wang A.P., Liao X.P., Zheng W.Y. The prevalence of tuberculosis in beef cattle farms in Yichun City, Jiangxi Province was detected by γ-interferon ELISA. *Animal Husbandry and Feed Science.* **2019,** *40(03)*, 104-107. **(in Chinese)**
103. Chen L.X. Epidemiological investigation of bovine tuberculosis in some areas of Jiyuan City, Henan Province. *China Dairy.* **2017,** *(05)*, 54-55. **(in Chinese)**
104. Yang X.C., Li K.H., Wu X.J., Tao T.G.S., Wang J. Comparative test of skin test method and γ-interferon ELISA method in tuberculosis quarantine of dairy cows. *Shanghai Journal of Animal Husbandry and Veterinary Medicine*. **2017**, *(05)*, 56-57,59. **(in Chinese)**
105. Shen W., Zhang Z.Q., Jiao Z.X., Zhou L., Zhang D.H. Quarantine of tuberculosis in a dairy farm in Shihezi area. *China Cattle Science.* **2006**, *(06)*, 84-85. **(in Chinese)**
106. Yang G.L., Wnag J., He X., Cui J.X., Dong S.F., Zhang H. Analysis report of bovine tuberculosis surveillance in Liaoning province in 2020. *Modern Journal of Animal Husbandry and Veterinary Medicine.* **2021**, *(09)*, 64-66. **(in Chinese)**
107. Salitanati J. The monitoring results and analysis of bovine tuberculosis in the surrounding areas of Urumqi, Xinjiang. *China Dairy.* **2016**, *(04)*, 55-56. **(in Chinese)**
108. Wu Y.W., Wang X.L., Zhang Y.L., Wnag Y.L., Li Z.H., Zhang X.J. γApplication of γ-interferon ELISA detection method in epidemiological investigation of bovine tuberculosis. *China Animal Health Inspection.* **2016,** *33(09)*, 80-82. **(in Chinese)**
109. Yang Y.H. Discussion on tuberculosis quarantine of dairy cattle in Liwo Town. *Agricultural*

Technical Services. **2016**, *(01)*, 177. **(in Chinese)**
110. Niu J.Q., Suolangquza, Xu Y.F., Labadunzhu, Li P. The comparative test of skin test and ELISA in the detection of tuberculosis in Linzhi cattle was compared. *Chinese Journal of Veterinary Medicine.* **2013**, *49(10)*, 35-37. **(in Chinese)**
111. Zhang X.H. Investigation and Control of Dairy Cow Tuberculosis in Yinchuan Area. *nongjia zhifu guwen.* **2015**, *(18)*, 48-49. **(in Chinese)**
112. Duan Z.T., Zhuang Y.Y. The application of SICT and ELISA in the detection of tuberculosis in dairy cows. *Zhejiang Journal of Animal Science and Veterinary Medicine.* **2015**, *40(05)*, 4-5. **(in Chinese)**
113. Pu C. Experimental study on the current situation of bovine tuberculosis infection in permanent village of Bayi Town, Linzhi City, Tibet. *Veterinary Orientation.* **2018**, *(14)*, 213. **(in Chinese)**
114. Xu X.Y., Xu Z.J., Wang X.Z., Zhang C.F., Chen C.H. Analysis of tuberculosis surveillance results of dairy cows in Jiangsu from 2007 to 2013. *China Animal Health Inspection.* **2014**, *31(07)*, 75-77. **(in Chinese)**
115. Liu J.W., Ren J.J., Xu J.P., He Z.B., Sun T., Li J., et al. Tianjin Dairy Cow Tuberculosis Purification Project. *China Animal Health Inspection.* **2014**, *31(08)*, 58-62. **(in Chinese)**
116. Jiang L. Dairy cattle tuberculosis detection and comprehensive prevention and control suggestions in Xichang City. *Livestock and Poultry Industry.* **2014**, *(10)*, 75. **(in Chinese)**
117. Song Z.B., Hou Y.X., Guo J.P. Clinical Study on Detection Technology of Dairy Cow Tuberculosis. *China Dairy Cattle.* **2019,** *(02)*, 19-21. **(in Chinese)**
118. Yang J.L., The current situation and prevention and control countermeasures of brucellosis and tuberculosis in dairy cows in Nanning City. *Hubei Journal of Animal and Veterinary Sciences.* **2013**, *34(03)*, 46-47. **(in Chinese)**
119. Xierenzuoma. Serological detection of cow tuberculosis in Gabasongdu Town, Tongde County. *Hubei Journal of Animal and Veterinary Sciences*. **2013.** *34(10)*, 19-20. **(in Chinese)**
120. Wang C.M., Huercha., Hu Z.X., Song Y.C., Liu L.Y. Bovine tuberculosis quarantine combined with intradermal allergy and interferon-γ ELISA. *Shanghai Journal of Animal Husbandry and Veterinary Medicine.* **2016**, *(02)*, 44-45. **(in Chinese)**
121. Fu S.G., Cao X,Z., Ma J.C., Wang X.L., Xia S.H., Zhao J,. Analysis of the results of delayed type hypersensitivity test in monitoring tuberculosis in Yinchuan dairy cows. *China Animal Health Inspection.* **2012**, *29(02)*, 36-37+64 **(in Chinese)**
122. Wang Y.M., Ying Q.X., Zheng L.Y., Lu H,L. Control of tuberculosis and brucellosis in large-scale dairy farms in Yanping District of Nanping City. *Fujian Journal of Animal Husbandry and Veterinary.* **2011**, *33(01)*, 11-12. **(in Chinese)**
123. Hao C.C., Shi J.H., Yao H.R. Investigation on Dairy Cow Tuberculosis in Hualong County. *Modern Agricultural Sciences and Technology.* **2010**, *(09)*, 324. **(in Chinese)**
124. Bai X.W. Investigation on Tuberculosis Infection of Dairy Cows in Qiabuqia Town of Gonghe County. *Chinese Qinghai Journal of Animal and Veterinary Sciences.* **2010,** *40(01)*, 25-26. **(in Chinese)**
125. Guo J., Huang M.S. Diagnosis and Experience of Beef Cattle Tuberculosis. *Hunan Journal of Animal Science & Veterinary Medicine*. **2010**, *(04)*, 26-27. **(in Chinese)**
126. Li S.S., Ma D.L. The detection report of tuberculosis in dairy cows in the big beach area. *The Chinese Livestock and Poultry Breeding.* **2009**, *5(06)*, 111-112. **(in Chinese)**
127. Wen F.Y., Wang G.L., Yu G.F. Discussion on the problems existing in the quarantine and purification of cow tuberculosis. *Beijing Agriculture.* **2009**, *(24)*, 49-51. **(in Chinese)**

128. Lan S.K., Li Z.Y., Wei K.J. Effect and experience of comprehensive prevention and control of cow tuberculosis from 1998 to 2007. *Animals Breeding and Feed.* **2008**, *(06)*, 42-45. **(in Chinese)**
129. Xin D., An N. Xinjiang Bole cow tuberculosis investigation. *XINJIANG XUMUYE.* **2007**, *(01)*, 38-39.
130. Liu Y., Hu X.T. Detection and Purification of Dairy Cow Tuberculosis in Yili Reclamation Area. *Chinese Journal of Animal Husbanday and Veterinary Medicine.* **2006**, *(01)*, 53-54. **(in Chinese)**
131. Qin P., Hu H.R., Analysis of monitoring results of bovine tuberculosis in Jin 'an District in 2005. *Modern Agricultural Science and Technology.* **2006**, *(05)*, 67. **(in Chinese)**
132. Fang J.Y., Huang G.M., Wei C.K., Liang H.B. Dairy buffalo tuberculosis quarantine report in Shanglin County. *Guangxi Journal of Animal Husbandry & Veterinary Medicine.* **2003**, *(04)*, 167-168. **(in Chinese)**
133. Sang C.X., Wang X.Q., Mulati., Zhang Q,Y., Ma J. Monitoring and prevention of bovine tuberculosis in Urumqi county. *XINJIANG XUMUYE.* **2012**, *(S1)*, 22-23. **(in Chinese)**
134. Wuniqiemu W. Epidemiological investigation of bovine tuberculosis in Hejing county. *XINJIANG XUMUYE.* **2012**, *(S1)*, 24-25. **(in Chinese)**
135. Qing Y.X., Li A.Q. Rural cow tuberculosis quarantine situation and prevention and control countermeasures. *Modern Agricultural Sciences and Technology.* **2011**, *(21)*, 331-332. **(in Chinese)**
136. Huang A.F., Wang X.M., Qi X.H., Wang X.H. Epidemiological investigation of bovine tuberculosis in Wuwei City. *Journal of Animal Science and Veterinary Medicine.* **2011**, *30(06)*, 33-34,37. **(in Chinese)**
137. Sheng Y., Fan F. Clinical application of γ-interferon test to detect bovine tuberculosis in Wuxi area. *Agricultural Development and Equipments.* **2015**, *(02)*, 70+74. **(in Chinese)**
138. Li C.Y., Investigation of bovine tuberculosis in Dulan County, Qinghai Province. *nongjia zhifu guwen.* **2018**, *(10)*, 74. **(in Chinese)**
139. Duan X.J., Zhang P., Wang B., Zhang S.X., Dang R.Y., Yang Z.Q. Investigation of bovine tuberculosis in Guanzhong area of Shaanxi province. *China Animal Health Inspection.* **2009**, *26(07)*, 50-52. **(in Chinese)**
140. Yang A.G., Lu Z.P., Hou W., Mo Q., Zhou M.Z., Yin J., et al. Epidemiological investigation of bovine tuberculosis in the main breeding areas of dairy cows in Sichuan Province from 2016 to 2017. *Heilongjiang Animal Science and Veterinary Medicine.* **2019**, *(10)*, 93-95. **(in Chinese)**
141. Dou S.Y., Cao L.P., Wang J., Zhou F., Cheng w.j. A comparative study on the detection of bovine tuberculosis by bovine tuberculin intradermal allergy test and γ-interferon ELISA test. *Journal of Animal Science and Veterinary Medicine.* **2014**, *33(06)*, 8-10. **(in Chinese)**
142. La H., Wang S.X., Wang X.Y., Ma R.L., Fu Y.J., Wang Z.F., et al. Monitoring and epidemic trend analysis of bovine tuberculosis in Qinghai Province from 2009 to 2014. *Heilongjiang Animal Husbandry and Veterinary Medicine.* **2015**, *(20)*, 91-93. **(in Chinese)**
143. Zhao Y.L. Dairy cow tuberculosis detection test. *Shandong Journal of Animal Science and Veterinary Medicine.* **2008**, *(05)*, 13 (**in Chinese**).
144. Jiao X.L. Surveillance and Control Measures of Bovine Tuberculosis in Qinghai Province. *Chinse Journal of Veterinary Sciences and Technology.* **2003**, *(07)*, 68-69. **(in Chinese)**
145. Guo H.Y., Gong B.T., Chen Y.W. Investigation of bovine tuberculosis in Hainan. *Chinse Journal of Veterinary Science and Technology.* **2002**, *(03)*, 17. **(in Chinese)**
146. Bai B., Xiao K., Jin Z.Q. Quarantine situation of bovine tuberculosis in Wudang District of Guiyang City. *Chinese Journal of Veterinary Medicine.* **2003**, *(02)*, 46. **(in Chinese)**

147. Cao XP, Yang YH, Li CH, Li XL. Detection report of cattle tuberculosis. *Contemporary Animal Husbandry.* **2005**, *(11)*, 15. **(in Chinese)**
148. Pan Y.X., Guo Y.Z. Monitoring and purification of bovine tuberculosis in Tengchong City. *Yunnan Journal of Animal Science and Veterinary Medicine.* **2017**, *(06)*, 13-14. **(in Chinese)**
149. Tian G.Z., Tian X.F., Zhou S.L. Epidemiological investigation of tuberculosis in dairy cows in Mudan District of Heze City. *Shandong Journal of Animal Science and Veterinary Medicine.* **2017**, *38(01)*, 54. **(in Chinese)**
150. Jin A.C,R. Investigation on bovine tuberculosis in Zhenqin Town, Chengduo County, Yushu Prefecture. *Chinese Qighai Journal of Animal and Veterinary Sciences.* **2015**, *45(03)*, 16. **(in Chinese)**
151. Gu S.S., Fam F. Epidemiological investigation of bovine tuberculosis in Wuxi City in recent years. *The Chinese Livestock and Poultry Breeding.* **2015**, *11(03)*, 3-5. **(in Chinese)**
152. Lei Y.L. Investigation on tuberculosis infection of dairy cows in Beishan Township of Menyuan County. *Journal of Animal Science and Veterinary Medicine.* **2014**, *33(03)*, 96,98. **(in Chinese)**
153. Luo S.H., Hong F. The present situation and prevention measures of cow tuberculosis in Changning County. *Yunnan Journal of Animal Science and Veterinary Medicine*. **2013**, *(02)*, 23-24. **(in Chinese)**
154. Xiong C.X., Xiong H.M. Monitoring and purification of bovine tuberculosis in Midu County of Yunnan Province. *China Animal Health Inspection.* **2011**, *28(01)*, 58-59. **(in Chinese)**
155. Zhang Z.L. Monitoring and Purification of Dairy Cow Tuberculosis in Linxia Prefecture. *Journal of Animal Sciaence and Veterinary Medicine.* **2011**, *30(04)*, 82-83. **(in Chinese)**
156. Chang M.H. Investigation on tuberculosis infection of dairy cows in Guinan area of Qinghai. *The Chinse Livestock And Poultry Breeding.* **2008**, *4(23)*, 69. (in Chinese)
157. Kuerban J., Chen Y.Q., Abulimiti T., Luo X. Investigation and Control of Bovine Tuberculosis in Wensu County. *XINJIANG XUMUYE.* **2007**, *(01)*, 37-38. **(in Chinese)**
158. Guan S.G., Zhang S.X., Li H.P., Wen S.Z., Yang L.H., Kang Y.Q. Experience and Understanding of Purification of Dairy Cow Brucellosis Tuberculosis. *Veterinary Orientation***2007**,.*(01)*, 24. **(in Chinese)**
159. He B.H., Xu Z.M., Liu J.P., Xie S.L. Dairy cattle tuberculosis monitoring report in Jingbian County. *Journal of Animal Science and Venerinary Medicine.* **2007**, *(04)*, 97-98. **(in Chinese)**
160. He C.J., , Bao S.K., Kong X.Y., Zhu J. Investigation of brucellosis tuberculosis infection in dairy cows in Haibei area. *Chinse Qinghai Journal of Animal and Veterinary Sciences.* **2006**, *(01)*, 27. **(in Chinese)**
161. Chen C.M., Chen M.Q. Investigation on an outbreak of tuberculosis in a dairy farm. *China Animal Health Inspection.* **2005**, *(05)*, 34. **(in Chinese)**
162. Li Z.W., Yan S.W., Xu X.Q., Fei Y.M., Han Y. Investigation on Dairy Cow Tuberculosis in Wulan County. *Chinse Qinghai Journal of Animal Science and Veterinary Medicine*. **2004**, *(02)*, 2. **(in Chinese)**
163. Wu Z.B., Zheng W. Report on the detection of brucellosis and tuberculosis in dairy cows in Huangyan District. *China Animal Health Inspection.* **2004**, *(01)*, 33. **(in Chinese)**
164. Mu A.X., Lu Y.G., Ying X.F., Gu G.L., Yang Z.Z., Tao Y. Detection results of tuberculosis and brucellosis in dairy cows in Hangzhou. *Zhejiang Journal of Animal Science and Veterinary Medicine.* **2003**, *28(03)*, 28-29. **(in Chinese)**
165. Liu D.Y., Zhou J.M., Yu W.C., Deng W.L., Zhang Z.S., Han X.L., Zhu G.Q.,Tian F.Y. Dairy cattle tuberculosis monitoring in Datong County. *Chinese Qinghai Journal of Animal and Veterinary*

Sciences. **2003**, *(01)*, 38. **(in Chinese)**
166.Zhao Y.J., Li Y.L. Investigation on Tuberculosis Quarantine of Dairy Cattle in Ledu County. *Chinese Qinghai Journal of Animal and Veterinary Sciences.* **2003**, *(06)*, 29. **(in Chinese)**
167.Liu D.X., Tan Z,X., Qiu L.X., Lu X.H., Wang C.J. Investigation of animal brucellosis and bovine tuberculosis in Hunan province.*Human Journal of Animal Science and Veterinary Medicine.* **2003**, *(04)*, 27-28. **(in Chinese)**
168.Shi Q.Y., Hu Y.J., Xiao Y., Li R. Investigation on Dairy Cow Tuberculosis in Minhe County. *Chinese Qinghai Journal of Animal and Veterinary Sciences.* **2003**, *(01)*, 17. **(in Chinese)**
169.Wang Y,Z., Niang X.J., Liu G.L. Dairy cattle tuberculosis monitoring report in Huangnan area. *Chinese Qinghai Journal of Animal and Veterinary Sciences.* **2003**, *(03)*, 43. **(in Chinese)**
170.Wang M.H. Bovine tuberculosis detection and existing problems. *Fujian Journal of Animal Husbandry and Veterinary Medicine.* **2002**, *(S1)*, 25. **(in Chinese)**
171.Li L.B, Qi M. Quarantine report of bovine tuberculosis in Mudanjiang city. *China Animal Health Inspection.* **2001**, *(06)*, 28-29. **(in Chinese)**
172.Chen Z.Q., Zhang L., Chen Y. Investigation on the infection of tuberculosis and brucellosis in dairy cows in Hami City. *China Animal Health Inspection.* **2001**, *(11)*, 34. **(in Chinese)**
173.Ying R.L., Yu Q.F., Bao W.H., Wang M.Y., Wang W.J. Report on the detection of cow tuberculosis in Ningbo City. *Zhejiang Animal Science and Veterinary Medicine.* **2001**, *(02)*, 28. **(in Chinese)**
174.Zheng R.Y., Luo P.F., Wang J.Q., Shu Z., Li A.Q., Zhao Y.K., et al. Comparison of three different test methods in the application of bovine tuberculosis detection. *Grass-feeding Livestock.* **2022**, *(02)*, 33-38. **(in Chinese)**
175.Gao Y.Y. Study on Dairy Cow Tuberculosis Monitoring in Jingbian County of Shaanxi Province. *Northwest A&F University*. **2018. (in Chinese)**
176.Shi M.J., Wang J.Z., Liu G., Cai B. Quarantine of cow tuberculosis in Xinghua City. *China Animal Health Inspection.* **2006**, *(02)*, 31-32. **(in Chinese)**
177.Tian H. Investigation on Epidemiology and Control of Dairy Cow Tuberculosis in Anding District of Dingxi City. *Gansu Agricultural University*. **2004. (in Chinese)**
178.Aini A., Qi J., Zheng X.H., Zhang L.R. Investigation of bovine tuberculosis in Shache County, Xinjiang Animal. *Animal Husbandry and Veterinary Medicine.* **2009**, *41(12)*, 106-107. **(in Chinese)**
179.Li X.S., Shen C., Xu Y.X., Yang F.L. Investigation and Control Measures of Dairy Cow Tuberculosis in Huangzhou District of Huanggang City. *Heilongjiang Animal Husbandry and Veterinary Medicine.* **2014**, *(22)*, 78-79. **(in Chinese)**
180.Huang J.J. Quarantine and Control of Bovine Tuberculosis and Brucellosis in Guangzhou. *Guangdong Journal of Animal and Veterinary Medicine.* **1997**, *(03)*, 27-28. **(in Chinese)**
181.Mo J.H.，He J.Q.，Wang H.Y. Lian F.，Xin B.X.，He Y.X.，Dong C.R.，Wu X.J. Analysis of Dairy Cow Tuberculosis Quarantine in Anyang City. *Henan Animal Husbandry and Veterinary.* **1995**, *(03)*, 50-51. **(in Chinese)**
182.Peng P.C. Investigation report of cow tuberculosis in Shaoguan city. *Guangdong Journal of Health and Epidemic Prevention.* **1991**, *(02)*, 74-76. **(in Chinese)**
183.Wu W.H., Song D.S., Tian M.L., Yang L., Yang M.S., Sun Q.Y., et al. Detection and purification of bovine tuberculosis in a dairy farm in Qingzhen City. *The Chinese Livestock and Poultry Breeding.* **2014**, *10(10)*, 94-95. **(in Chinese)**
184.Feng L.H., Feng S .Z., Pu J.P., Li J.L., Kuai S.X., Zhu L.L, et al. Brucellosis and tuberculosis surveillance process. *China Animal Husbandry and Veterinary Medicine.* **2007**, *(02)*, 156-158. **(in**

Chinese)
185. Yuan L.G., Li J., Pu J.W., Li Y., Qi Y.Y. Epidemiological investigation of main infectious diseases of yak in Tianshan area. *China Herbivore Science.* **2015**, *35(03)*, 37-38. **(in Chinese)**
186. Yao J.X., Liu F.N., Zhu Y. Investigation and Analysis of Main Diseases of Cattle in Dingxi City of Gansu Province. *China Animal Health Inspection.* **2018**, *35(03)*, 10-13. **(in Chinese)**
187. Chen F.M., Cheng G.M., Ma A.X., Hu S.L. Serological investigation of BVD-MD, IBR and TB in dairy cows in Weifang and surrounding areas. *Heilongjiang Journal of Animal Science and Veterinary Medicine.* **2016**, *(18)*, 114-117. **(in Chinese)**
188. Zhang S., Yang A.G., Hou W., Guo L., Zhang L., Yang X.L., et al. Investigation on tuberculosis infection in dairy farms in Hongya County of Sichuan Province in 2018. *Parasitoses and Infectious Diseases.* **2022**, *20(04)*, 190-194. **(in Chinese)**
189. Jiang J.H., Dai G.W., Wei B.W., Xie L.H., Liang Z., Li M., et al. Epidemiological investigation on main epidemic diseases of dairy cows in Wuzhou City from 2003 to 2013. *Journal of Animal Science and Veterinary Medicine.* **2014**, *33(05)*, 99-101. **(in Chinese)**
190. Bu Q. Investigation on tuberculosis monitoring status of yak cows in pasture of Qumalai County. *Chinese Qinghai Journal of Animal and Veterinary Sciences.* **2006**, *(05)*,24. **(in Chinese)**
191. Yuan F.Y. Liu Q.C. Analysis on the Current Situation and Control Countermeasures of ' Two Diseases ' of Dairy Cows in Jimsar County. *XINJIANG XUMUYE.* **2009**, *(06)*, 47-48. **(in Chinese)**
192. Wang K.F., Yuan H.X., Zhai G.P. Analysis and Discussion on a Case of Suspected Transfer of Animals under Legal Isolation. *Chinese Journal of Animal Husbandry and Veterinary Medicine.* **2012**, *(01)*, 35. **(in Chinese)**
193. Liao Y.F. Diagnosis and prevention and control of sheep tuberculosis. *Chinese Journal of Animal Husbandry and Veterinary Medicine.* **2015**, *(11)*, 53-54. **(in Chinese)**
194. Zhao A.L., Gong X.M. Thoughts and Countermeasures of Two Cases of Sheep Tuberculosis. *Chinese Journal of Animal Husbandry and Veterinary Medicine.* **2005**, *(08)*, 51. **(in Chinese)**
195. Ran M.T., Yu B., Tan S.W., Wang F., Ai Y.P. Establishment and preliminary application of PCR detection method for goat tuberculosis. *The Chinese Livestock and Poultry Breeding.* **2013**, *9(06)*, 103-105. **(in Chinese)**
